# The cyclic di-GMP receptor YcgR links the second messenger with the putrescine quorum sensing system in modulation of *Dickeya oryzae* motility

**DOI:** 10.1128/mbio.01016-25

**Published:** 2025-05-30

**Authors:** Weihan Gu, Yufan Chen, Congcong Xie, Zhongqiao Chen, Huagui Gao, Yaping Zhu, Lian-hui Zhang, Lisheng Liao

**Affiliations:** 1 Guangdong Province Key Laboratory of Microbial Signals and Disease Control, Integrative Microbiology Research Center, South China Agricultural University, Guangzhou, China; 2 Research Center of Chinese Herbal Resource Science and Engineering, Key Laboratory of Chinese Medicinal Resource From Lingnan, Ministry of Education, Joint Laboratory of National Engineering Research Center for the Pharmaceutics of Traditional Chinese Medicines, Guangzhou University of Chinese Medicine, Guangzhou, China; 3 School of Biological and Environmental Engineering, Jingdezhen University, Jingdezhen, China; University of Washington School of Medicine, Seattle, Washington, USA

**Keywords:** *Dickeya oryzae*, c-di-GMP, putrescine, bacterial motility, YcgR

## Abstract

*Dickeya oryzae* is a prevalent pathogen capable of infecting a variety of crops and plants, and its cell motility plays a key role in invasion of host and subsequent systemic infection. We recently demonstrated that the bacterial second messenger c-di-GMP and the putrescine (PUT)-mediated quorum sensing (QS) system are, respectively, involved in negative and positive regulation of bacterial motility, and vice versa, biofilm formation. In this study, we aimed to investigate the potential interaction of these two signaling mechanisms in the modulation of bacterial motility. The results indicated that null mutation of the PUT system did not seem to have much effect on the cellular level of c-di-GMP; however, deletion of the genes encoding c-di-GMP degradation led to a significant reduction in PUT production. A subsequent study unveiled that the second messenger signaling system interacted with the putrescine QS system through the c-di-GMP receptor YcgR. This interaction enhanced the activity of SpeA, which is the rate-limiting enzyme in the putrescine biosynthesis pathway, resulting in increased intracellular putrescine level. Critically, this facilitative effect was inhibited by c-di-GMP molecules; thus, SpeA, YcgR, and c-di-GMP constitute a regulatory loop modulating *D. oryzae* motility by controlling the rate of putrescine biosynthesis. The findings from this study provide the first insight into the interaction mechanism between c-di-GMP and putrescine signaling systems in bacteria.

**IMPORTANCE:**

*Dickeya oryzae* is an important bacterial pathogen that can infect numerous plants and crops, leading to substantial economic losses, especially in rice and banana cultivation. Bacterial motility is a crucial pathogenic factor for *D. oryzae* as it enables the pathogen to compete for food resources and invade host plants. This motility is negatively regulated by the second messenger c-di-GMP and positively regulated by the quorum sensing signal putrescine (PUT). However, the potential connection between c-di-GMP and PUT signaling systems in regulating the motility of *D. oryzae* has not been understood. Here, we reveal the link and mechanism of the interaction between them, demonstrating that c-di-GMP interacts with the PUT system via its receptor YcgR. The significance of our research lies in providing the first insight into the molecular interaction between c-di-GMP and PUT signaling networks, both of which are widely conserved signaling mechanisms, and sheds light on the complex and sophisticated regulatory mechanisms that govern bacterial motility and virulence.

## INTRODUCTION

Cell motility is an important mechanism for bacterial pathogens to compete for food resources and infect their host organisms. To survive in harsh environmental conditions, bacteria evolved mechanisms allowing them to switch rapidly between planktonic motile and sessile non-motile forms in response to environmental changes. The state of attachment facilitates the adherence of bacterial cells to host tissues and enhances resistance against the immune response of host organisms (1, 2), whereas the planktonic state, featured by strong motility, is not only important for successful invasion of the host but also critical for avoidance of host defense responses, colonization, and systemic infection (3–5). Bacterial motility is normally closely linked to chemotaxis, which in combination enables bacteria to detect/pursue nutrients and to settle in their preferred colonial ecological niches (6). Bacterial motility is mainly mediated by flagella and controlled by flagella quantity, direction, and speed of rotation (7). The research progress over the years has demonstrated that bacterial cell motility is an important pathogenic factor, and the bacteria with impaired motility are significantly less pathogenic than the wild-type parental strains (8, 9).

Polyamines are a group of biologically active polycationic compounds found in a wide variety of living organisms. The most common polyamines in microorganisms are putrescine, spermidine, and spermine, with putrescine being the central product of the polyamine synthesis pathway (10). In bacteria, there are two main pathways for the synthesis of putrescine, i.e., the ornithine pathway and the arginine pathway. In the ornithine pathway, ornithine decarboxylase (SpeC) catalyzes the formation of putrescine from ornithine. In the arginine pathway, arginine decarboxylase (ADC), encoded by the *speA* gene, catalyzes the conversion of arginine to agmatine, which then converts into putrescine by agmatinase (11). Spermidine and spermine are produced by the reaction of putrescine and aminopropyl, catalyzed by spermidine synthase and spermine synthase, respectively (12, 13). In general, polyamines are associated with many cellular processes in bacteria, such as growth under anaerobic conditions, iron carrier synthesis, peptidoglycan synthesis, biofilm formation, bacterial motility, and resistance to oxidative stress and antibiotics (14–18). In recent years, there has been increasing evidence that polyamines are an intraspecies and interkingdom cell-cell quorum sensing signal playing a key role in the regulation of bacterial physiology and virulence (19, 20). Among them, putrescine is primarily involved in the regulation of bacterial motility and biofilm formation (9). However, the molecular mechanism of putrescine in modulation of bacterial motility and biofilm formation remains poorly understood.

Cyclic di-GMP (c-di-GMP) is a widespread second messenger in bacterial species, whose homeostasis is controlled by diguanylate cyclases (DGCs) responsible for c-di-GMP biosynthesis and phosphodiesterases (PDEs) involved in c-di-GMP degradation. Specifically, DGCs have a GGDEF domain, and PDEs contain either an EAL or a HD-GYP domain (21–23). The second messenger has been shown to regulate a number of physiological processes, such as biofilm formation, motility, virulence, and cell differentiation and multiplication, through various downstream receptors or effector proteins (24, 25). Among them, the PilZ structural domain proteins constitute the most widely distributed c-di-GMP receptor family, which can be classified into the single PilZ structural domain class, YcgR class, BcsA class, and Alg44 class (26). YcgR was one of the first PilZ structural domain proteins discovered and well characterized. It consists of the N-terminal YcgR domain and the C-terminal PilZ domain. YcgR binds c-di-GMP molecules via the PilZ domain and then regulates bacterial biological functions through protein-protein interactions or protein-DNA interactions (26). Previous studies have shown that in *Escherichia coli*, c-di-GMP promotes the interaction of YcgR with the flagellar proteins FilG and FilM to inhibit bacterial motility by modulating the direction and rate of flagellar rotation (27). In *Klebsiella pneumoniae*, c-di-GMP promotes the binding of MrkH, a YcgR-class protein, to the promoters of *mrkHI* and *mrkA*, which encode the transcriptional regulators associated with the regulation of type 3 fimbriae expression and biofilm formation, respectively (28–30).

*Dickeya oryzae* is the causative agent of bacterial foot rot disease in rice crops, leading to severe economic losses (31, 32). The pathogen was initially known as *Erwinia chrysanthemi* pv. *zeae* and reclassified as *D. zeae* of the new genus *Dickeya* in 2005 (33). More recently, it was renamed as *D. oryzae* (34). The pathogen is capable of producing a range of pathogenic factors, including the phytotoxin zeamines (35), extracellular enzymes (36), and type III effectors (37, 38). Bacterial motility is one of the key virulence determinants of *D. oryzae,* which plays a vital role in its colonization and invasion on rice seeds (8, 9, 39). Recently, several regulatory mechanisms that modulate bacterial motility have been unveiled in *D. oryzae* EC1 (40), including the acyl-homoserine lactone (AHL) QS system (32), the polyamine-mediated QS and pathogen-host communication system (9), and the c-di-GMP signaling system (8). Among them, putrescine together with its transporters PotF and PlaP positively regulates the motility of strain EC1 (9), whereas the accumulated c-di-GMP negatively regulates the bacterial motility through its downstream receptor protein YcgR (8).

To explore the potential link between putrescine and c-di-GMP signaling systems in the regulation of *D. oryzae* motility, in this study, we validated the impact of two signaling systems on the bacterial motility under the same experimental conditions. We then tested the cellular level of putrescine in *D. oryzae* wild-type strain EC1 and its mutants defective in c-di-GMP degradation and a functional YcgR receptor. The results showed that the putrescine level was significantly reduced in these mutants compared to the wild-type strain EC1. *In vitro* protein interaction and *in vivo* bacterial two-hybrid assays revealed a direct interaction between YcgR and SpeA, a key rate-limiting enzyme of the putrescine synthesis pathway. Subsequently, we tested whether YcgR could promote SpeA activity to increase putrescine production and the impact and consequences of c-di-GMP on the YcgR-SpeA interaction. Furthermore, we also tested the relative roles of c-di-GMP and putrescine QS system in the regulation of *D. oryzae* cell motility. The findings depict a novel mechanism with which a negative- and a positive-regulatory system acts in a coordinated manner to control the transition between the motile and non-motile bacterial status in response to environmental changes.

## RESULTS

### Putrescine and c-di-GMP play opposite roles in the regulation of *D. oryzae* swimming motility

**Fig 1:**
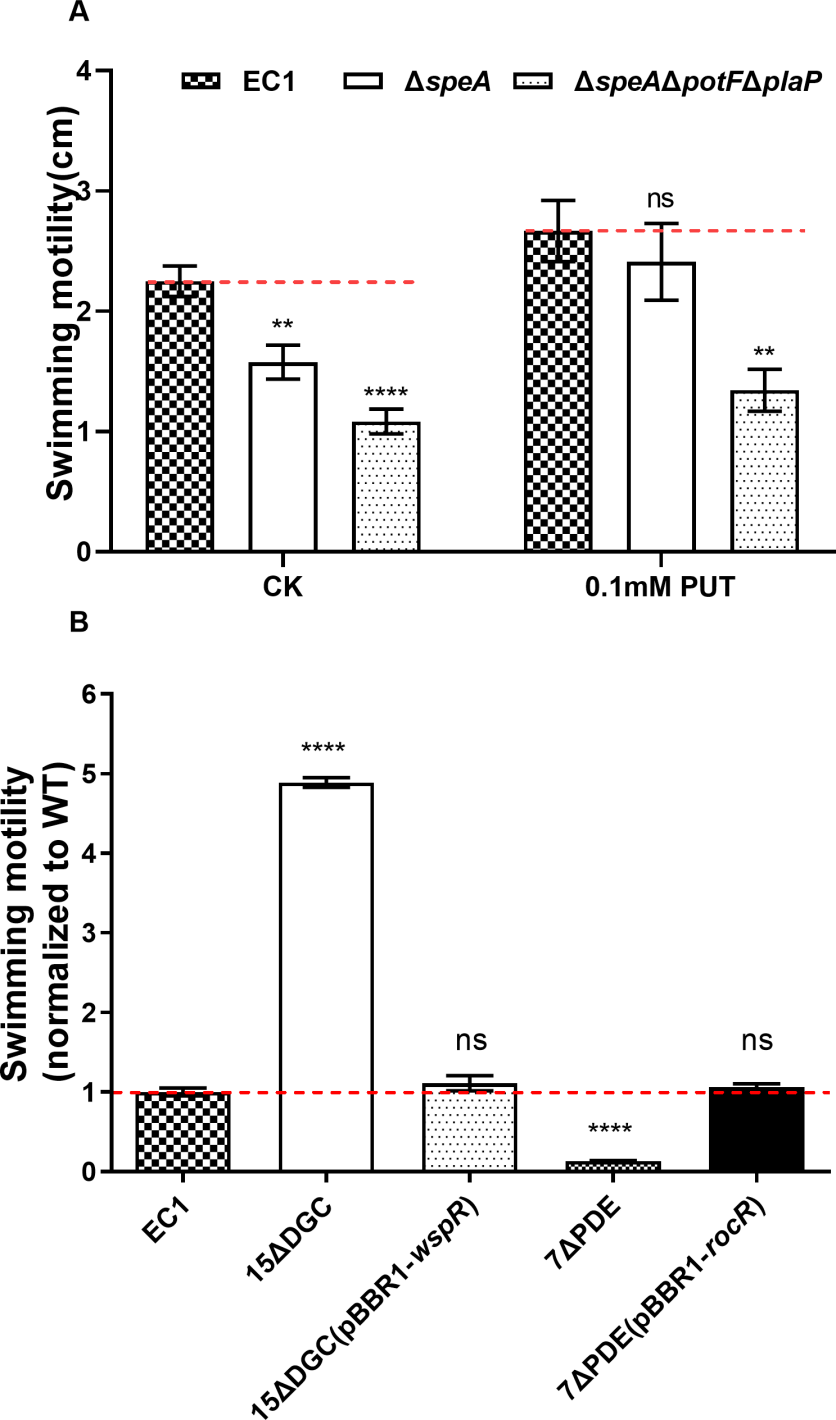
Second messenger c-di-GMP and PUT QS system play a negative and positive role, respectively, in the regulation of *D. oryzae* swimming motility. (**A**) Swimming motility of *D. oryzae* EC1 and its derivatives defective in PUT biosynthesis (*speA*) and transportation (*potF*, *plaP*) in the absence or presence of 0.1 mM PUT. (**B**) Swimming motility of *D. oryzae* EC1 and its derivatives defective in c-di-GMP metabolism, including mutant 15∆DGC with all the c-di-GMP synthase genes being deleted and mutant 7∆PDE with all the c-di-GMP degradation genes being knocked out. The well-characterized DGC gene *wspR* and PDE gene *rocR* from *P. aeruginosa* were used in heterologous complementation analysis, as indicated. Dotted lines indicate the swimming motility of the wild-type level. The data shown are the mean ± standard deviations (*n* = 3). Statistics significance: ****, *P* < 0.0001; **, *P* < 0.01; ns, *P* > 0.05 (by one-way ANOVA with multiple comparisons).

To explore the potential cross-talking between putrescine and c-di-GMP signaling pathways, which were shown previously to be associated with the regulation of bacterial motility (10, 11), we conducted experiments to compare the regulatory patterns of these two signaling mechanisms in modulation of bacterial motility under the same experimental conditions. Null mutation of *speA* in *D. oryzae* strain EC1*,* which encodes a rate-limiting enzyme in the putrescine synthesis pathway, decreased the swimming motility by about 30% compared to the wild-type EC1 (Fig. 1A). Exogenous addition of 0.1 mM putrescine rescued the swimming motility of Δ*speA* (Fig. 1A), validating the role of putrescine in the regulation of bacterial motility. This was further confirmed by the deletion of the two putrescine-specific transporter genes, i.e., *potF* and *plaP* (10), in the mutant Δ*speA*. The resultant triple mutant Δ*speA*Δ*potF*Δ*plaP* showed much reduced swimming motility, which was at about 40% of the wild-type level and could not be fully restored by exogenous addition of putrescine (Fig. 1A). The second messenger c-di-GMP seemed to exert a much stronger influence on the *D. oryzae* motility than putrescine. When all the c-di-GMP synthase coding genes were knocked out in strain EC1, the mutant 15ΔDGC showed drastically enhanced swimming motility, which was up to about 480% of the wild-type level. Conversely, the mutant 7ΔPDE with all the genes encoding c-di-GMP-degrading enzymes being deleted became nonmotile (Fig. 1B). Complementation of the mutants 15ΔDGC and 7ΔPDE with previously characterized heterologous DGC and PDE genes (*wspR; rocR*) (41, 42), respectively, fully restored their swimming ability to the wild-type levels (Fig. 1B). These results validated that the presence of the PUT signal exerts a positive effect in the regulation of *D. oryzae* swimming motility, whereas the presence of the second messenger c-di-GMP plays a negative role in the modulation of the bacterial motility.

### c-di-GMP accumulation in *D. oryzae* decreases the PUT cellular level

**Fig 2:**
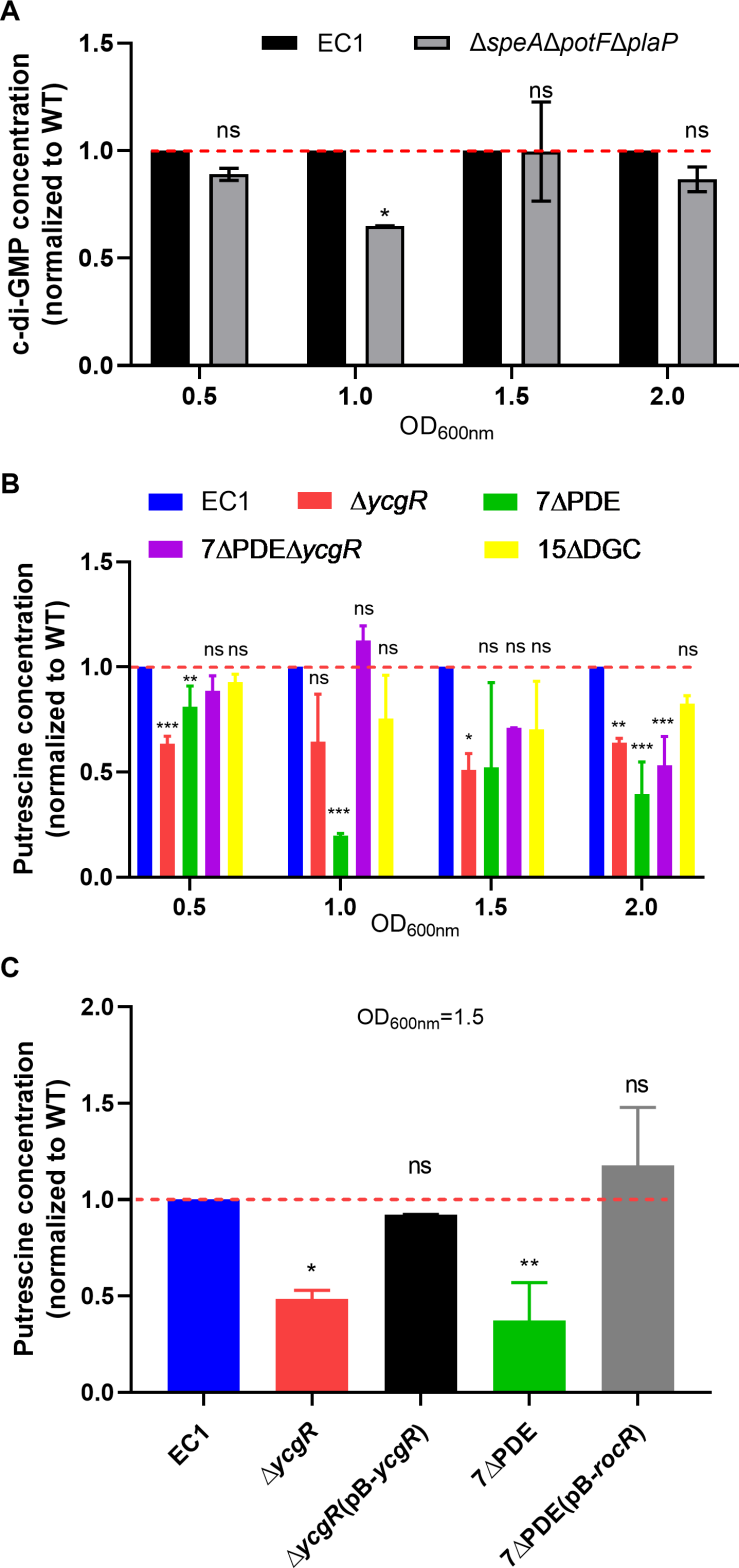
Cellular c-di-GMP and putrescine levels in *D. oryzae* EC1 and its derivatives. (**A**) Quantitative measurement of cellular cyclic di-GMP levels of wild-type strain EC1 and mutant ∆*speA*∆*potF*∆*plaP* defective in PUT biosynthesis and transportation in MM. The dotted line indicates the wild-type c-di-GMP level. The data shown are the mean ± standard deviations (*n* = 3). Statistical significance: *, *P <* 0.05; ns, *P* > 0.05 (by Student’s unpaired *t* test). (**B**) Quantitative measurement of the cellular putrescine concentration of strain EC1 and derivatives defective in c-di-GMP synthesis (15∆DGC), degradation (7∆PDE), and signal transduction (∆*ycgR*) in MM. The data shown are the mean ± standard deviations (*n* = 3). Statistical significance: ****, *P* < 0.0001; ***, *P* < 0.001; **, *P* < 0.01; *, *P* < 0.05; ns, *P* > 0.05 (by one-way ANOVA with multiple comparisons). The dotted line indicates the wild-type PUT level. (**C**) Quantitative measurement of the cellular putrescine concentration of strain EC1, ∆*ycgR*, ∆*ycgR*(pB*-ycgR*)*,* 7∆PDE, and 7∆PDE(pB-*rocR*) in MM. The data shown are the mean ± standard deviations (*n* = 3). Statistical significance: **, *P* < 0.01; *, *P* < 0.05; ns, *P* > 0.05 (by one-way ANOVA with multiple comparisons).

To understand how the PUT QS system and the c-di-GMP signaling mechanism could act together to coordinate the transition between motile and nonmotile lifestyles of *D. oryzae*, the cellular levels of PUT and c-di-GMP were measured in the wild-type strain EC1 and its corresponding mutants, including the mutant 7ΔPDE with all the genes encoding c-di-GMP-degrading enzymes being deleted and the deletion mutant of *ycgR* that encodes a c-di-GMP receptor (43). Intracellular PUT and c-di-GMP contents were determined by liquid chromatography-mass spectrometry (LC-MS) assays. Since tryptone and yeast extract of LB medium interfere with c-di-GMP quantification (42), minimal medium (MM) was used for assaying c-di-GMP content in bacteria. Considering that the double-deletion mutant Δ*speA*Δ*speC,* in which both putrescine biosynthesis genes *speA* and *speC* of arginine and ornithine pathways, respectively, were deleted, exhibited a significantly slower growth rate in MM than its parental wild-type strain EC1 (Fig. S1), we selected the mutant Δ*speA*Δ*potF*Δ*plaP,* which lacks PUT synthase SpeA and transporters PotF and PlaP (9) to investigate the impact of putrescine deficiency on c-di-GMP biogenesis. In addition, given that PUT is a QS signal and that QS signal production is commonly cell density-dependent (20, 32), it would be useful to investigate the impact of PUT deficiency on c-di-GMP metabolism at different growth stages. Results showed that in MM, there was no significant change in the c-di-GMP level in the mutant Δ*speA*Δ*potF*Δ*plaP* compared to wild-type EC1 under most growth periods (Fig. 2A), suggesting that c-di-GMP metabolism is not substantially influenced by the PUT signaling system. In contrast, in the same medium, the PUT concentration was found to decrease significantly by over 50%, 50%, and 30% in the mutants Δ*ycgR,* 7ΔPDE, and 7ΔPDEΔ*ycgR,* respectively, compared with wild-type EC1 when bacterial growth reached a high population density (OD_600_ = 1.5) (Fig. 2B). The findings suggest that the accumulated cellular c-di-GMP in the mutants 7ΔPDE and 7ΔPDEΔ*ycgR* may play a role in downregulation of PUT biosynthesis, and intriguingly, the absence of the c-di-GMP receptor YcgR seemed to have a similar effect as the accumulated c-di-GMP. Consistent with the above-speculated role of c-di-GMP in the regulation of PUT biosynthesis, there was no significant change in the cellular PUT content in the c-di-GMP null mutant 15ΔDGC compared to the wild-type EC1 under most growth periods (Fig. 2B).

To validate the above findings, the wild-type effector gene *ycgR* and the *rocR* gene encoding a well-characterized c-di-GMP degradation enzyme (42) were in *trans* expressed in the mutants Δ*ycgR* and 7ΔPDE, respectively, and their effects on PUT production were determined. The results showed that the production level of PUT in the complemented strains Δ*ycgR*(*ycgR*) and 7ΔPDE(*rocR*) was restored to the level of the wild-type strain (Fig. 2C), indicating that the c-di-GMP signaling mechanism plays a key role in the modulation of PUT biosynthesis. Similar results were also found when strain EC1 and its derivatives were cultured in LB medium (Fig. S2).

### Defect in PUT signaling results in decreased expression of the genes encoding c-di-GMP biosynthesis and its receptor

**Fig 3:**
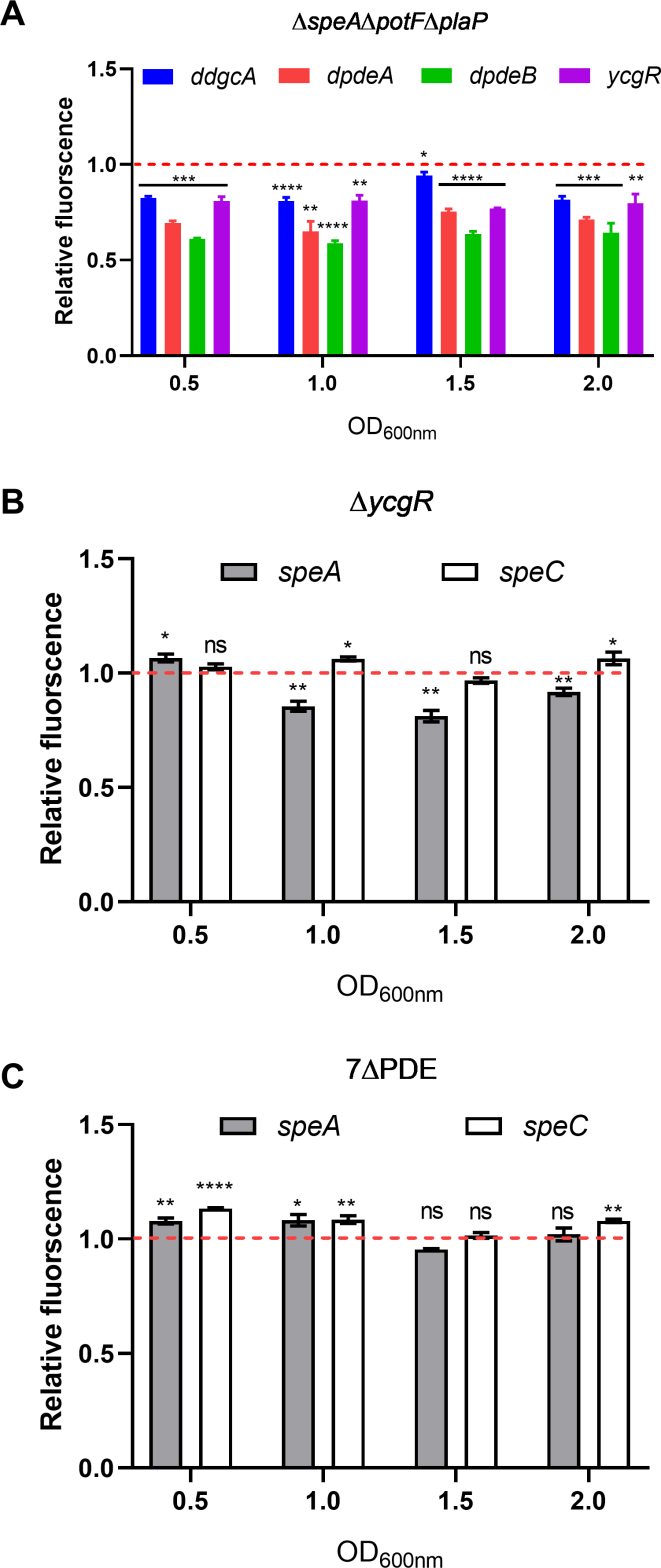
Transcriptional fusion assay of the genes encoding the c-di-GMP signaling system and PUT QS system in wild-type strain EC1 and corresponding mutants. (**A**) Transcriptional fusion assay of c-di-GMP system-related genes in wild-type strain EC1 and mutant ∆*speA*∆*potF*∆*plaP*. The following genes were quantified in this experiment: *ycgR*, encoding a PilZ domain c-di-GMP receptor; *ddgcA* (*W909_14945*), encoding the major diguanylate cyclase that synthesizes c-di-GMP; *dpdeA* (*W909_14950*), encoding the major phosphodiesterase that degrades c-di-GMP; *dpdeB* (*W909_10355*), encoding major bifunctional enzymes that can both synthesize and degrade c-di-GMP (8). (**B** and **C**) Transcriptional fusion assay of *speA* and *speC* encoding two key rate-limiting enzymes for putrescine synthesis in mutants ∆*ycgR* and 7∆PDE, respectively, compared to wild-type EC1. The bacterial cells were cultured in MM. The data shown are the mean ± standard deviations (*n* = 3). Statistical significance: ****, *P* < 0.0001; ***, *P* < 0.001; **, *P* < 0.01; *, *P* < 0.05; ns, *P* > 0.05 (by Student’s unpaired *t* test). The dotted line indicates the expression levels of these genes in the wild-type strain EC1.

To understand how c-di-GMP could modulate PUT biosynthesis, we first determined the transcript levels of *ycgR* and three genes associated with the c-di-GMP metabolism (*ddgcA, dpdeA,* and *dpdeB*) by transcriptional fusion assay. Among them, *ddgcA* (*W909_14945*) encodes the c-di-GMP synthase DdgcA, *dpdeA* (*W909_14950*) encodes the c-di-GMP degradation enzyme DpdeA, and *dpdeB* (*W909_10355*) encodes the bifunctional enzyme DpdeB, which account primarily for the dynamic changes of c-di-GMP content in *D. oryzae* as well as the motility phenotypic changes (8). The transcriptional expressions of these genes were assessed in wild-type strain EC1 and mutant Δ*speA*Δ*potF*Δ*plaP* in MM. The results showed that the expression levels of these c-di-GMP metabolism and signal transduction-associated genes in the mutant Δ*speA*Δ*potF*Δ*plaP* were decreased compared to the wild-type EC1 (Fig. 3A). Specifically, the expression levels of the four genes, *ddgcA*, *dpdeA*, *dpdeB*, and *ycgR*, in Δ*speA*Δ*potF*Δ*plaP,* were decreased by about 20%, 30%, 40%, and 20%, respectively, compared with the wild-type EC1 at the four time points of bacterial growth. It is interesting to note that PUT appeared to generate more or less equal impact on the transcriptional expression of the four genes associated with the c-di-GMP signaling system.

### c-di-GMP accumulation in *D. oryzae* has a minor effect on the transcriptional expression of PUT biosynthesis genes

Similarly, expression levels of *speA* and *speC*, which are critical for PUT biosynthesis (9), were assessed in wild-type strain EC1 and mutants Δ*ycgR* and 7ΔPDE in MM. The results showed that the two PUT synthase genes displayed less than 25% difference at the transcript level in mutants Δ*ycgR* and 7ΔPDE compared to the wild-type EC1 under most growth periods (Fig. 3B and C). The transcriptional analysis data did not seem to be agreeable with the results of the above-described biochemical assay, which showed that deletion of the genes encoding c-di-GMP biosynthesis or its receptor led to a significant reduction in cellular PUT level (Fig. 2B and C). These findings motivated us to test whether c-di-GMP and its receptor could interact with the PUT QS system at the post-transcriptional level.

### c-di-GMP receptor YcgR interacts with the proteins involved in PUT biosynthesis

**Fig 4:**
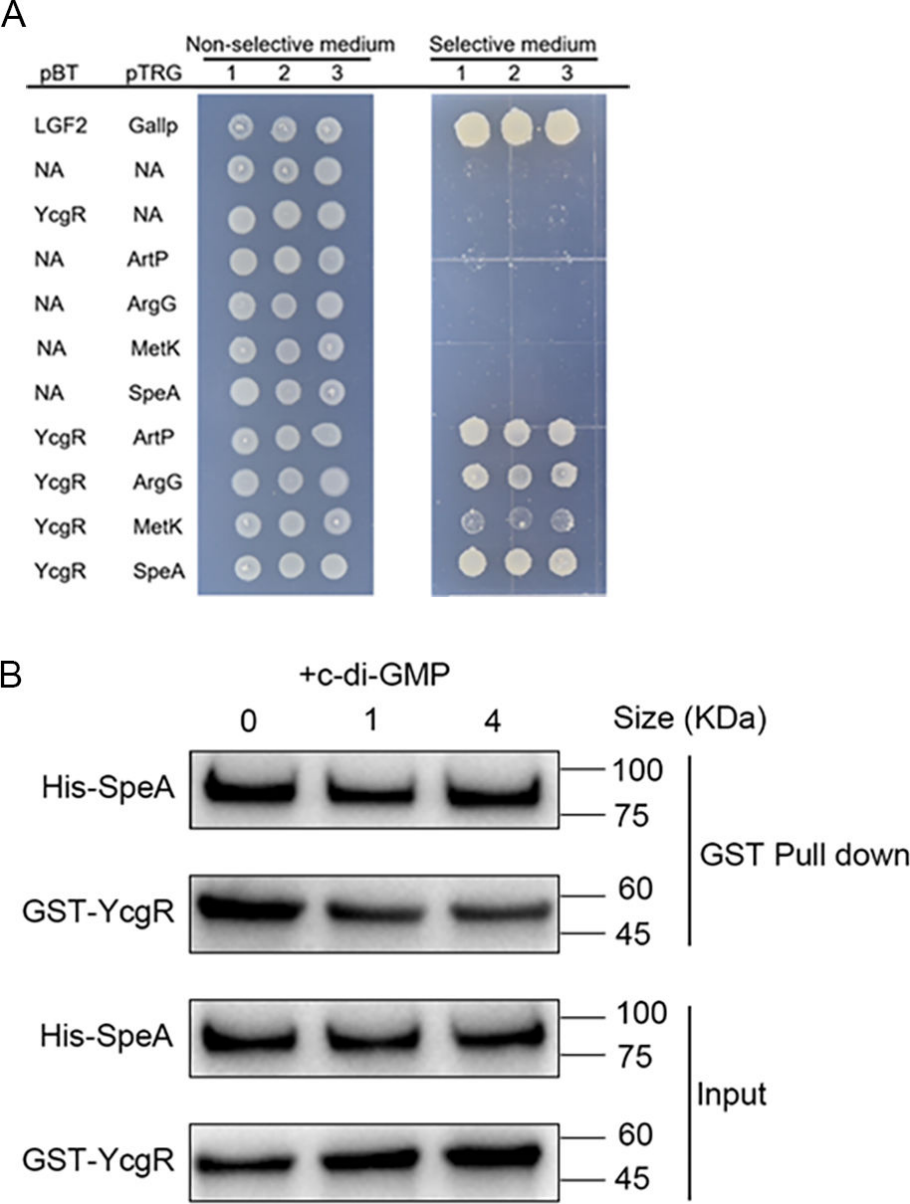
Identification of proteins potentially interacting with YcgR. (**A**) Identification of YcgR-interacting proteins by bacterial two-hybridization. Four proteins that directly interact with YcgR have been identified as follows: SpeA, biosynthetic arginine decarboxylase; ArtP, arginine ABC transporter ATP-binding protein; ArgG, argininosuccinate synthase; and MetK, S-adenosylmethionine synthase. (**B**) GST pull-down analysis of the effect of c-di-GMP on the YcgR-SpeA interaction. c-di-GMP was added in 0, 1, and 4 molar ratios respectively to YcgR, as indicated in the pull-down assay.

Given that both PUT and c-di-GMP signaling systems are involved in the regulation of *D. oryzae* motility, and that c-di-GMP is dependent on its downstream receptor YcgR to modulate the bacterial motility and biofilm formation (43) (Fig. S3), we decided to explore the potential interaction of YcgR with the proteins associated with PUT signal generation and transduction. Using immunoprecipitation-mass spectrometry (IP-MS) and bioinformatic analysis, seven proteins in the PUT biosynthesis pathway that could interact with YcgR were identified (Table 1). Among them, four proteins were validated by bacterial two-hybrid assay (B2H), namely, SpeA, ArgG, ArtP, and MetK (Fig. 4A). Specifically, SpeA catalyzes the synthesis of agmatine from L-arginine (9); ArgG catalyzes the synthesis of L-arginino-succinate acid from citrulline (44); ArtP is a transporter protein for facilitating the influx of extracellular L-arginine molecules (45); and MetK catalyzes the synthesis of S-adenosylmethionine from L-methionine (46), which further reacts with PUT to form spermidine. From the results of B2H, the proteins that interact most strongly with YcgR are revealed to be SpeA and ArtP, followed by ArgG and MetK (Fig. 4A). Further pull-down experiments validated that YcgR and SpeA could interact with each other *in vitro,* and their binding *in vitro* was not affected by c-di-GMP (Fig. 4B). Swimming motility assay of the mutants lacking these four proteins showed that only Δ*speA* was significantly less motile compared to the wild type, while the mutants Δ*argG*, Δ*artP,* and Δ*metK* displayed a comparable motility with the wild-type EC1 (Fig. S4). The results of the biofilm formation assay for these mutants also indicated that only Δ*speA* showed a significant increase in biofilm production compared with the wild type, while the other mutants did not show substantial differences compared with the wild type (Fig. S4).

**TABLE 1:** The proteins interacting with YcgR identified by Co-IP and protein profile analysis

Gene	ORF	Protein	Unique Pep Count
*argG*	*W909_00315*	Argininosuccinate synthase	3
*argA*	*W909_04780*	Amino-acid acetyltransferase	1
*artP*	*W909_05770*	Arginine ABC transporter ATP-binding protein	1
*plaP*	*W909_08760*	Putrescine/spermidine ABC transporter	1
*strP*	*W909_10855*	Polyamine ABC transporter permease	1
*speA*	*W909_17465*	Arginine decarboxylase	4
*metK*	*W909_17475*	S-adenosylmethionine synthase	9

Interestingly, previous reports showed that in several other bacterial species, including *Escherichia coli, Pseudomonas aeruginosa,* and *Salmonella,* YcgR-like Pilz proteins could interact with the bacterial flagellar proteins FliM, FliG, and MotC and thus influence the bacterial motility (27, 47, 48). However, in contrast, B2H and pull-down analyses did not support any potential interaction between YcgR with these flagellar protein homologs in *D. oryzae* EC1 (Fig. S5). These findings suggest that YcgR-like PilZ proteins could influence bacterial motility through different mechanisms in a bacterial species-dependent manner.

### Addition of YcgR enhances the enzymatic activity of SpeA *in vitro*

**Fig 5:**
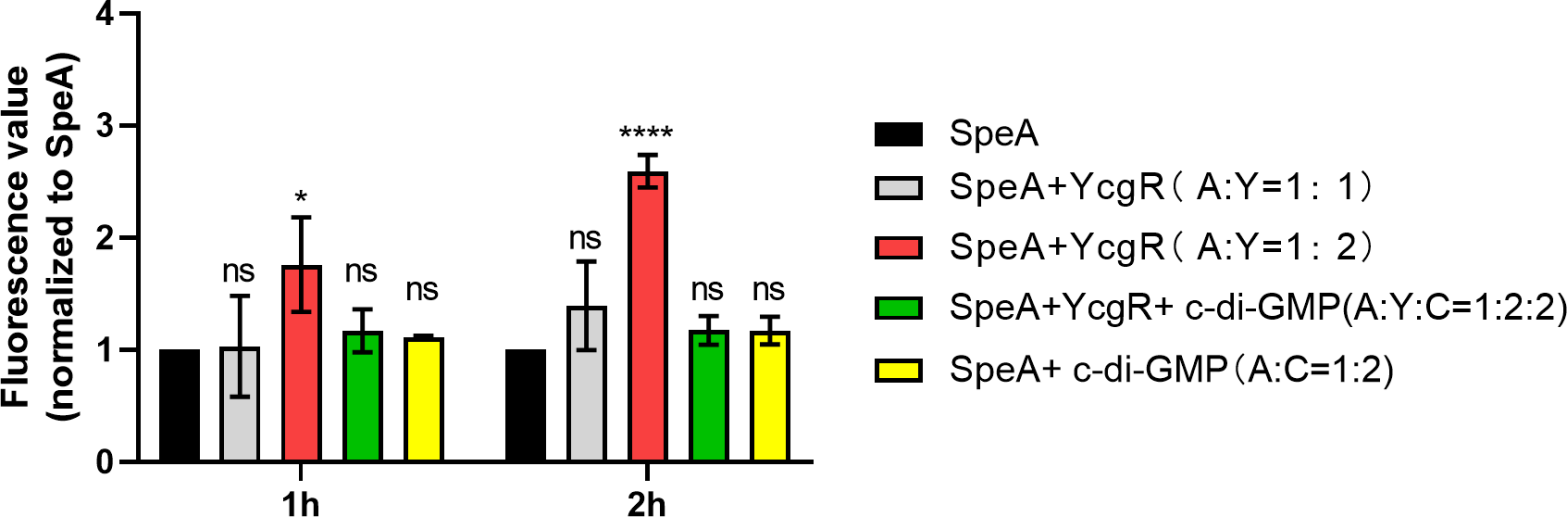
Addition of YcgR promotes SpeA enzymatic activity. SpeA enzymatic activity in the absence or presence of YcgR in different ratios, as indicated. The product agmatine was extracted at 1 h or 2 h after the initiation of the reaction, and its absorbance value was measured. The data shown are the mean ± standard deviations (*n* = 3). Statistics significance: ****, *P* < 0.0001; *, *P* < 0.05; ns, *P* > 0.05 (by Student’s unpaired *t* test).

Considering that SpeA is a key enzyme for PUT synthesis and that deletion of *speA* resulted in decreased swimming motility and increased biofilm formation in strain EC1 (Fig. S4), we therefore compared the enzymatic activity of SpeA in the presence or absence of YcgR. In *D. oryzae* EC1, SpeA catalyzes the conversion of arginine to agmatine, which in turn is converted into PUT in a two-step reaction (9). In this study, the concentration of agmatine was measured using the previously described *o*-phthalaldehyde (OPA) spectrofluorometric method (49, 50). Results showed that the addition of YcgR to the SpeA reaction system at a 2:1 ratio could significantly increase the production of agmatine compared to the control without YcgR (Fig. 5). The promoting effect of YcgR became more obvious with the increase in reaction time. Specifically, the yield of agmatine was increased by 75% compared to the null-YcgR group at 1 h post-addition of YcgR, which was increased by about 160% at 2 h post-interaction with YcgR (Fig. 5).

Given that either lacking YcgR or c-di-GMP degradation enzymes, which means substantially higher levels of accumulated c-di-GMP, could lead to much reduced PUT production, we speculated that c-di-GMP might counteract the facilitating activity of its receptor YcgR on PUT production. In previous studies, we have shown that YcgR binds c-di-GMP in a 1:1 molar ratio (43). Therefore, the same molar ratio of c-di-GMP as YcgR was added to the SpeA:YcgR (1:2 ratio) and SpeA reaction mixtures. Results showed that the addition of c-di-GMP did not seem to affect the SpeA activity, whereas it neutralized the promoting effect of YcgR on SpeA activity in the production of agmatine (Fig. 5).

### c-di-GMP plays a major role in regulating bacterial motility in comparison with PUT

**Fig 6:**
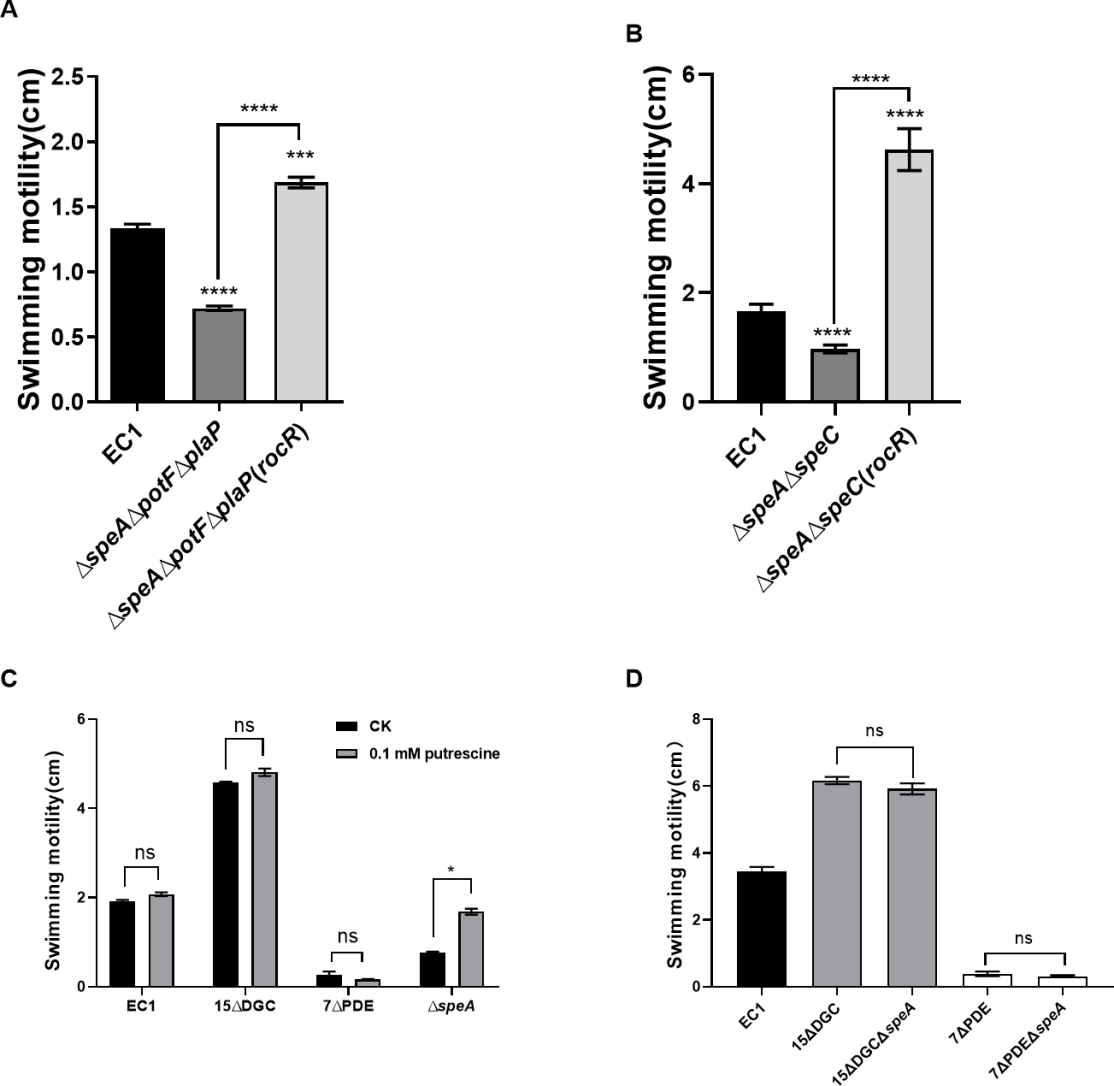
Relative strength of c-di-GMP and PUT signaling systems in regulation of bacterial motility. (**A**) Expression of *rocR* encoding c-di-GMP degradation restored and boosted the swimming motility of PUT signaling-defective mutant ∆*speA*∆*potF*∆*plaP*. The data shown are expressed as the mean ± standard deviations (*n* = 3). Statistical significance: ****, *P* < 0.0001; ***, *P* < 0.001 (by one-way ANOVA with multiple comparisons). (**B**) Expression of *rocR* encoding c-di-GMP degradation restored and boosted the swimming motility of fully PUT biosynthesis pathway-deficient mutant ∆*speA*∆*speC*. The data shown are expressed as the mean ± standard deviations (*n* = 3). Statistical significance: ****, *P* < 0.0001; ns, *P* > 0.05 (by one-way ANOVA with multiple comparisons). (**C**) Exogenous addition of PUT (0.1 mM) restored the swimming motility of mutant ∆*speA* but did not seem to affect the motility of strain EC1 and c-di-GMP mutants 15∆DGC and 7∆PDE. The data shown are expressed as the mean ± standard deviations (*n* = 3). Statistical significance: *, *P* < 0.05; ns, *P* > 0.05 (by Student’s unpaired *t* test). (**D**) Deletion of PUT synthase gene *speA* in c-di-GMP synthesis mutant 15∆DGC and degradation mutant 7∆PDE failed to generate significance on the bacterial swimming motility. The data shown are expressed as the mean ± standard deviations (*n* = 3). Statistical significance: ns, *P* > 0.05 (by one-way ANOVA with multiple comparisons).

The results in Fig. 1 showed that *D. oryzae* motility is positively regulated by the PUT QS system and negatively modulated by the second messenger c-di-GMP. It is intriguing which signaling system may play a dominant role in the modulation of bacterial motility. To address this point, we first expressed the *rocR* gene from *P. aeruginosa* in the PUT-deficient mutant Δ*speA*Δ*potF*Δ*plaP.* The *rocR* gene encodes a well-characterized PDE enzyme (42). Therefore, its expression in the mutant Δ*speA*Δ*potF*Δ*plaP* will generate a cellular environment deficient in both PUT and c-di-GMP molecules. The results showed that RocR could reverse the motility of mutant Δ*speA*Δ*potF*Δ*plaP* to a level higher than that of wild-type EC1 (Fig. 6A). To test whether RocR overexpression might lead to increased PUT production and consequently facilitate bacterial motility, we quantified the PUT contents, but the results ruled out this possibility as no significant difference was detected between strains Δ*speA*Δ*potF*Δ*plaP* and Δ*speA*Δ*potF*Δ*plaP*(*rocR*) (Fig. S7). Similarly, when *rocR* was overexpressed in the double-mutant Δ*speA*Δ*speC* with both PUT synthase genes of arginine and ornithine pathways respectively being deleted, the motility of Δ*speA*Δ*speC*(*rocR*) remained significantly enhanced compared to that of Δ*speA*Δ*speC* and even the wild type (Fig. 6B). We then tested whether the addition of PUT to the mutants 15ΔDGC and 7ΔPDE could affect their phenotypes in motility. The results showed that exogenous addition of QS signal PUT at a final concentration of 0.1 mM could restore the swimming motility of Δ*speA* to the wild-type level, whereas the QS signal failed to generate any significant impact on the motility phenotypes of both mutants 15ΔDGC and 7ΔPDE (Fig. 6C). Third, we determined whether deletion of the *speA* gene in the genetic background of the mutants 15ΔDGC and 7ΔPDE could influence their motility, but no significant changes were observed (Fig. 6D). Taken together, given that only the c-di-GMP metabolic enzyme could affect the phenotype of PUT-defective mutants and PUT was unable to change the altered motility of c-di-GMP-defective mutants, we conclude that the c-di-GMP signaling pathway occupies a dominant position in the regulation of bacterial motility compared to the PUT QS system.

## DISCUSSION

**Fig 7:**
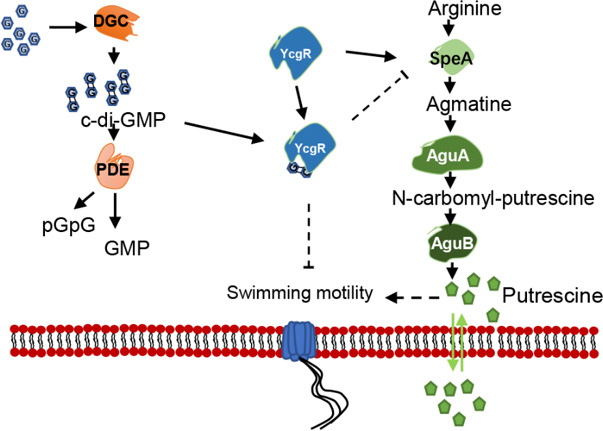
c-di-GMP and PUT QS system co-regulation model on *D. oryzae* motility. SpeA, arginine decarboxylase; AguA, agmatine deiminase; AguB, N-carbamoylputrescine aminotransferase; YcgR, a PilZ domain receptor for c-di-GMP. In this model, at low c-di-GMP levels, YcgR interacts with SpeA to facilitate PUT signal production and thus promote bacterial motility and planktonic lifestyle; on the other hand, upon reaching a threshold level, c-di-GMP competes for YcgR and forms the YcgR-c-di-GMP complex to suppress bacterial motility and switch to biofilm lifestyle.

PUT QS system and c-di-GMP second messenger signaling system are widely conserved regulatory mechanisms (8–10), and both of them are involved in modulation of bacterial motility and biofilm formation in *D. oryzae* (8, 9). Among them, the mechanism by which c-di-GMP negatively regulates bacterial motility has been elucidated. Specifically, when YcgR binds to c-di-GMP, it downregulates the expression of the flagellar master regulatory genes *flhCD*, which in turn reduces the expression levels of flagellar-related genes and consequently inhibits flagella biogenesis and flagella-dependent bacterial motility (43) (Fig. 7). However, how these two signaling mechanisms coordinate the transition between motile and nonmotile life states remains unknown. Given that these two signaling mechanisms are involved in the modulation of the same phenotypes, we speculated that PUT and c-di-GMP signaling mechanisms could interact and thus coordinate bacterial motility and biofilm formation. To unlock this mystery, we validated that PUT and c-di-GMP signaling systems play key roles in positive and negative regulation of *D. oryzae* motility, respectively (Fig. 1). We found that a high cellular level of c-di-GMP or deletion of its receptor protein YcgR, which is a PilZ-family protein, could cause a significant decrease in PUT biosynthesis (Fig. 2B and C), and vice versa, absence of PUT affected the expression of genes encoding c-di-GMP metabolism and receptor YcgR (Fig. 3A), suggesting a pattern of mutual modulation. Subsequently, we demonstrated that c-di-GMP receptor YcgR interacts physically with and promotes the catalytic activity of the PUT synthase SpeA (Fig. 4 and 5). We found that c-di-GMP could inhibit the formation of the SpeA-YcgR complex (Fig. 5), thus resulting in decreased PUT production. Different from previous reports, YcgR-like Pilz proteins regulate motility through a direct interaction with the bacterial flagellar motor proteins including FliM, FliG, and MotC (27, 47, 48). This study demonstrated that c-di-GMP receptor YcgR could interact with the key PUT synthase to promote bacterial motility. This finding adds a new entry to the functional list of c-di-GMP receptors.

Our previous studies demonstrated that the QS signal PUT produced by its synthase SpeA, together with its transporters PotF and PlaP, positively regulates the motility of strain EC1 (9), whereas the accumulated c-di-GMP, which is governed mainly by three major c-di-GMP metabolic enzymes, i.e., DdgcA, DpdeA, and DpdeB, negatively regulates the bacterial motility through its downstream receptor protein YcgR (8). To understand how PUT and c-di-GMP systems could interact in modulation of bacterial motility, in this study we first investigated whether these two systems could exert an influence on each other. We confirmed that the QS signal PUT positively regulates the cell motility of *D. oryzae* (Fig. 1A; Fig. S4A) but negatively controls the bacterial biofilm formation (Fig. S4B). Deletion of the genes encoding PUT signal biosynthesis and transportation resulted in decreased expression of the three major c-di-GMP metabolic genes (Fig. 3A); however, it did not seem to generate a significant impact on the cellular level of c-di-GMP (Fig. 2A). In contrast, deletion of the c-di-GMP receptor gene *ycgR* and all the seven genes encoding c-di-GMP degradation, including *dpdeA* and *dpdeB,* resulted in a much reduced cellular level of PUT (Fig. 2B and C). These findings suggest that the c-di-GMP signaling system may play a regulatory role over the PUT system in modulation of bacterial motility and biofilm formation. The notion was further endorsed by our findings that only the c-di-GMP metabolic enzyme could affect the phenotype of PUT-defective mutants, whereas the PUT signal was unable to change the altered motility of c-di-GMP-defective mutants (Fig. 6).

Although lines of evidence are accumulating that both c-di-GMP and polyamine signaling systems are associated with modulation of bacterial motility and biofilm formation (8, 47, 51), it is not yet clear whether and, if yes, how these two signaling systems could interact and regulate the same phenotypes in microorganisms. Given that YcgR plays a significant role in positive modulation of PUT signal production, we set out to screen for potential proteins that could physically interact with YegR and showed that it was able to interact with four PUT biosynthesis pathway-related proteins, especially SpeA, the rate-limiting enzyme of the PUT biosynthesis pathway (Fig. 4). *In vitro* experiments showed that YcgR could promote the enzymatic activity of SpeA by enhancing the production of agmatine (Fig. 5), which inevitably results in an increase in intracellular PUT level (51). This result is highly agreeable with the observation that mutant Δ*ycgR* was defective in PUT biosynthesis (Fig. 2C). Similarly, we found that the accumulation of c-di-GMP in mutant 7ΔPDE caused a drastic decrease in PUT production (Fig. 2C), and the addition of c-di-GMP molecules to the enzyme reaction system of SpeA and YcgR neutralized the promoting effect of YcgR on agmatine production (Fig. 5). Given that YcgR is the receptor of c-di-GMP (27), one plausible reasoning is that high levels of c-di-GMP preferentially bind to YcgR and thus prevent it from interacting with and abolishing its facilitative effect on the catalytic activity of SpeA.

Additionally, an intriguing phenomenon is that the PUT production in the mutant Δ*ycgR* decreased compared to the wild-type strain EC1, yet its motility was significantly enhanced relative to the EC1 (Fig. 2; Fig. S3). This might be due to the fact that the deletion of YcgR not only affected the intracellular PUT production but also impaired the response to c-di-GMP. Our findings given in Fig. 6 demonstrate that c-di-GMP plays a dominant role in regulating the motility of EC1, partially explaining why the motility of the Δ*ycgR* mutant is significantly enhanced relative to the wild-type strain. Subsequently, when we knocked out and overexpressed *ycgR* in the mutant Δ*speA*, the motility of the strains significantly increased and decreased compared to Δ*speA* (Fig. S6). This indicates that even in the absence of SpeA, a key enzyme for PUT biosynthesis, YcgR still negatively regulates the motility of EC1.

Taken together, the data from this study demonstrate that c-di-GMP could interact with the PUT QS system via a PilZ domain receptor YcgR, which enhances PUT production by promoting the enzymatic activity of SpeA, and this facilitative effect could be inhibited by c-di-GMP molecules. Therefore, SpeA, YcgR, and c-di-GMP metabolic enzymes constitute a regulatory loop in the modulation of *D. oryzae* motility, as well as biofilm formation and bacterial virulence, by coordinating the rate of PUT biosynthesis and c-di-GMP metabolism (Fig. 7). Such an interactive mechanism between two signaling systems would allow *D. oryzae* to tap on an extra signal input to boost cell motility when necessary and facilitate invasion and systemic infections.

## MATERIALS AND METHODS

### Bacterial strains and plasmids

Bacterial strains and plasmids used in this research are listed in Table S1 in the supplemental material. *Escherichia coli* was routinely grown at 37°C in Luria-Bertani (LB) medium. *D. oryzae* EC1 and its derivatives were grown at 28°C in LB medium, as previously reported. MM agar plates were used for conjugation (32). The following antibiotics were added at the indicated final concentrations when required: ampicillin (Amp) at 100 µg/mL, kanamycin (Km) at 50 µg/mL, streptomycin (Str) at 50 µg/mL, and polymyxin (Pm) at 30 µg/mL. The optical density at 600 nm (OD_600_) of the bacterial culture was measured by using a NanoDrop 2000c system (Thermo Fisher Scientific, USA).

### Mutant construction and complementation

Generation of in-frame gene deletion mutants was performed using the suicide vector pKNG101 and triparental mating according to a previously described protocol (8). In-frame deletion of the coding regions of all genes was done by the allelic-exchange method (8). Flanking regions of each coding region were amplified by PCR using the specific primers listed in Table S2 in the supplemental material. A complementation assay was performed by using the plasmid pBBR1-MCS4 and triparental mating, according to a previously described protocol (8). The coding region of the target gene was amplified by PCR using specific primers (Table S2).

### Bacterial motility assay

Bacterial swimming motility was assessed in a semisolid medium plate with 0.25% agar (per liter containing 10 g tryptone, 5 g yeast extract, 10 g NaCl, and 2.5 g agar). Fresh bacterial culture grown to an OD_600_ of 1.0 (1 µL) was spotted on the center of the plate and incubated at 28°C for 12 to 18 h before measurement.

### Biofilm formation assay

Biofilm formation assay was performed as described previously (8). In brief, bacterial cells grown to an OD_600_ of 2.0 were inoculated in glass test tubes with SOBS (Super Optimal Broth with 1% sucrose) medium for 24 h and then stained with 0.1% (wt/vol) crystal violet. Biofilm mass was quantified by measuring the absorbance at 570 nm after decolorization with a mixed solution of 80% ethanol and 20% acetic acid.

### Quantitative analysis of c-di-GMP by liquid chromatography-mass spectrometry

Quantification of c-di-GMP levels in wild-type strain EC1 and mutant Δ*speA*Δ*potF*Δ*plaP* was done based on the method described previously (8). Cellular c-di-GMP concentration was determined by perchloric acid lysis and liquid chromatography-mass spectrometry (LC-MS) in wild-type EC1 and mutants defective in the PUT QS system. The samples were separated by using a Syncronis C18 column (Thermo Fisher Scientific, USA) fitted with a 100- by 2.1 mm guard column with a flow rate of 0.2 mL/min, and the cycle time was 10 min. c-di-GMP was detected with an Orbitrap mass analyzer on the Q Exactive Focus system (Thermo Fisher Scientific, USA) in the positive ionization mode. c-di-GMP levels were normalized to the total protein per milliliter of the culture. Data represent the means from three independent cultures, with error bars indicating the standard deviations.

### Quantitative analysis of PUT by LC-MS

Quantification of PUT levels in wild-type strain EC1 and mutants was done based on the methods described previously with minor modifications (52–55). In brief, cells were grown overnight in LB medium at 28°C, adjusted to an OD_600_ of 1.0, and then subcultured in 15 mL MM with a 100-fold dilution in a 50 mL culture tube (Crystalgen, USA). When the bacterial culture was grown to an OD_600_ of 0.5, 1.0, 1.5, and 2.0, an aliquot of 2 mL was transferred into a 15 mL centrifuge tube. Bacterial cells were then collected by centrifugation at 4,000 rpm for 5 min, and 2 mL xTractor Buffer (Clontech, TaKaRa Biomedical Technology [Beijing] Co., Ltd., China) was added to each tube to lyse the bacterial cells. Then, 800 µL of the lysed sample was then taken in a new 15 mL centrifuge tube. For each sample, derivatization was carried out by adding 100 µL of benzoyl chloride and 1 mL of 2 M NaOH and vortexed for 2 min and incubated for 20 min at 37°C. Two milliliters of saturated sodium chloride solution was added to each tube and vortexed for 2 min, followed by adding 2 mL of petroleum ether for extraction. The samples were extracted overnight at 4°C and then centrifuged at 4,000 rpm for 5 min at 4°C using a 5810 R fixed-angle rotor centrifuge (Eppendorf, Germany). One milliliter of the upper layer of petroleum ether was dried under vacuum in a new 2 mL tube, followed by addition of 500 µL of methanol. After filtering the organic solvent through a 0.22 µm filter, all samples were stored at −20°C. Benzoylated PUT was used as a standard for analysis with high-performance liquid chromatography mass spectrometry (LC-MS). The derivatized samples were separated by using an ACQUITY UPLC HSS T3 column (Waters, Milford, MA, USA) fitted with a 100 × 2.1 mm guard column with a flow rate of 0.3 mL/min. The Q Exactive Focus system (Thermo Fisher Scientific) was used to verify the identity of each peak observed in HPLC fractions. A standard curve was generated by using various concentrations of benzoylated PUT in duplicates.

### Construction of fluorescence reporter strains and fluorescence analysis

The promoter region of the genes in Fig. 3 was amplified using the primers listed in Table S2 and ligated into the promoterless *gfp*-reporter plasmid pPROBE-NT (56) for generation of the constructs such as p*ddgcA*_gfp_. These constructs were separately mobilized into wild-type EC1 and the mutants described in Fig. 3 by triparental mating with the helper strain HB101(pRK2013) to construct fluorescence reporter strains. The reporter strains were cultured in MM or LB medium, and samples were collected, as indicated, for monitoring the fluorescence on a BioTek SYNEGRY H1. The relative fluorescence was expressed as the fluorescence monitored at specific time points normalized to the fluorescence of wild-type strain EC1.

### Protein expression and purification

The coding sequence of *speA* was amplified by PCR using *D. oryzae* EC1 genomic DNA as the template and then cloned into the pET-32a(+) expression vector at the BamHI and HindIII sites using a ClonExpress II one-step cloning kit (Vazyme Biotech Co., Ltd., China). The coding sequence of *ycgR* was amplified in the same way and then cloned into the pGEX-6P-1(+) expression vector at the XhoI and BamHI sites. Recombinant plasmids were respectively transformed into *E. coli* DH5α competent cells for sequencing, and the correct constructs of pET-speA and pGEX-ycgR were respectively transformed into *E. coli* BL21(DE3) for fusion protein expression. For overexpression of fusion proteins, cultures grown overnight were inoculated into 200 mL LB medium in a 500 mL Fernbach flask and grown with shaking until the OD_600_ reached ~0.6 to 0.8 prior to adding isopropyl-β-D-thiogalactopyranoside (IPTG) to a final concentration of 0.5 mM to induce fusion protein expression, and cultures were placed in a shaker at 16°C grown overnight with shaking. Cells were harvested by centrifugation at 4,000 rpm for 20 min at 4°C and resuspended in 20 mL xTractor buffer (Clontech, TaKaRa Biomedical Technology [Beijing] Co., Ltd., China) for lysis. Cell suspensions were incubated at room temperature for 10 min with gentle shaking. Crude lysates were centrifuged at 4,000 rpm for 90 min at 4°C, and supernatants were filtrated using 0.45 µm syringe filters (Pall Corporation). Affinity purification was performed at 4°C using Talon metal affinity resins (Clontech, TaKaRa Biomedical Technology [Beijing] Co., Ltd., China).

For the fusion protein SpeA, the resin and the column were washed with 10 column volumes of equilibration buffer (50 mM sodium phosphate, 300 mM sodium chloride, and 20 mM imidazole, pH 7.4) before adding the lysate supernatant. The fusion protein was eluted with gradient elution buffer (50 mM sodium phosphate, 300 mM sodium chloride, 50/100/150/200/300 mM imidazole, pH 7.4). For the fusion protein YcgR, the resin and the column were washed with 10 column volumes of equilibration buffer (1× PBS buffer [pH 7.4]) before adding the lysate supernatant. The target protein was eluted with elution buffer (10 mM reduced glutathione, 50 mM Tris-HCl, pH 7.4). The protein concentration was determined at 280 nm using the NanoDrop 2000c system (Thermo Fisher Scientific, USA) after staining with Coomassie brilliant blue.

### Enzymatic activity assay

The enzymatic activity of SpeA was measured according to a procedure described previously with minor modifications (57). The reaction mixture consisted of 5.767 × 10^−4^ µM SpeA protein and 0.1 µg L-arginine in 0.5 mL reaction buffer (50 mM HEPES, 2.5 mM MgSO_4_, 3.3 mM DTT, 0.5 mM PLP, pH 8.0). YcgR was added in molar ratios of 1:0, 1:1, 1:2, and 2:1 respective to SpeA, and c-di-GMP was added in molar ratios of 2:1, 1:1, 1:2, and 1:4 respective to YcgR. The mixture was incubated in a water bath at 37°C for 2 h, and aliquots of 200 µL were taken out every 1 h. The reaction was stopped by adding 200 µl NaCl-saturated 10% KOH solution, and then 200 µL n-butanol was added for the extraction of agmatine. After 8 h extraction, 20 µL of the top n-butanol layer was taken and mixed with 200 µL o-phthalaldehyde (OPA) reagent (0.8 mg/mL of OPA, pH10) to derivatize agmatine. Quantification of agmatine was performed by measuring the fluorescence of derivatized agmatine at excitation 340 nm and emission at 460 nm on a BioTek SYNEGRY H1.

### Immunoprecipitation-mass spectrometry (IP-MS) and bioinformatic analysis

Cells of strain EC1 (GFP-YcgR) were harvested by centrifugation and washed twice with PBS buffer and resuspended in PBS to a final OD_600_ = 4.0. The cross-linker DTBP (Thermo Scientific, catalog no. 20665) was added to the cell suspension at a final concentration of 5 mM and incubated for 45 min at room temperature. The reaction was stopped by adding Tris-HCl to a final concentration of 20 mM and mixed gently for 15 min. The cell lysates of strain EC1 (GFP-YcgR) were prepared by using the French Pressure cell (100 Mpa, one passage) with 20 U/mL DNase I and proteinase inhibitor cocktail (Thermo Scientific, catalog no. 87786). These nondenatured lysates were subject to immunoprecipitation with GFP-Trap (Chromo Tek, gta-20). The IP proteins (about 30  µg) were digested in solution using trypsin for 20  h at 37°C, and the digested peptides were subjected to Nano-HPLC/ESI-ion trap-MS/MS analysis with a Q Exactive mass spectrometer (Thermo Fisher Scientific, US). Raw MS data files were processed and analyzed using Mascot 2.2 (Matrix Science, UK) for database search with the following parameters: Database, *Dickeya_zeae*_NZ_ CP006929.1; enzyme, trypsin; variable modifications, oxidation (M); fixed modifications, carbamidomethyl (C); missed cleavages, 2; peptide mass tolerance, 20 ppm; fragment mass tolerance, 0.1 Da; filter by score, ≥20.

### Bacterial two-hybrid assay

Bacterial two-hybrid assay to assess the presence of protein-protein interactions between YcgR and PUT pathway-associated proteins was conducted as previously described (58), by using the BacterioMatch II two-hybrid system kit (Stratagene, San Diego, CA). In this assay, approximately 50 µg of each plasmid harboring pBT-*ycgR* and pTRG-*artP*/*argG*/*metK*/*speA* was co-transformed into the host strain, XL1-Blue MRF' Kan, through chemical transformation after adding 1 mL of the preheated LB medium in each tube. The cells were then incubated at 37°C with shaking at 200 rpm for 2 h. Thereafter, the LB medium was removed through centrifugation, and the cells were resuspended in 1 mL of M9 + His-dropout broth and incubated at 37°C with shaking at 200 rpm for another 2 h. Co-transformation mixtures were then grown on nonselective (NSM; M9 + His-deficient) and selective (SSM; M9 + His-deficient + 5 mM 3-amino-1,2,4-triazole) screening media for 24–36 h at 37°C. Notably, incubation was conducted for an additional 24–36 h at 30°C in the dark, for the weak interactors. Colonies that grew on both the NSM and SSM plates were identified as positive interaction pairs, whereas the colonies observed only on NSM but not on SSM were indicative of a negative interaction between the proteins.

### Pull-down assay

The *in vitro* pull-down assay to assess the protein-protein interaction between YcgR and SpeA was conducted as previously described by using the Pierce GST Protein Interaction Pull-Down Kit (Thermo Fisher) (58). In brief, GST-YcgR was used as the bait, and SpeA-His was used as the prey. For the binding assays, 100 to 150 µg of GST-YcgR was immobilized into 50 µL glutathione agarose resin, and then the same molar ratio of prey protein SpeA-His was added to the same column for capture. c-di-GMP was added according to the molar ratio of YcgR to c-di-GMP of 1:1, 1:2, and 1:4. After washing with the wash solution, protein complexes were eluted with 10 mM reduced glutathione and analyzed by 10% SDS-PAGE gel and Western blot following the standard protocols.

### Bacterial growth analysis

Bacterial cultures grown overnight in LB medium were adjusted to the same cell density and added in MM at a 1:100 ratio. Two hundred microliters of the diluted culture was added to each well and grown at 28°C with low-intensity shaking, and the growth curve was analyzed by the Bioscreen-C automated growth curve analysis system (OY Growth Curves AB, Ltd., Finland).

### Statistical analysis

All experiments were individually performed at least twice in three replicates each time. Data shown are expressed as the means from three replicates, and error bars indicate the standard deviations. Statistical comparison was performed by using Student’s unpaired *t* test or one-way ANOVA with multiple comparisons in GraphPad Prism 8.0 software (GraphPad, La Jolla, CA).

## Data Availability

The genome sequence of *D. oryzae* EC1 is accessible in the NCBI database under accession no. NZ_CP006929.1. The nucleotide sequences of *wspR* and *rocR* in *P. aeruginosa* PAO1 are accessible in the NCBI database under gene IDs 878337 and 878871, respectively.

## SUPPLEMENTAL MATERIAL

Supplemental material mbio.01016-25-s0001.docx Supplemental tables and figures.

ASM does not own the copyrights to Supplemental Material that may be linked to, or accessed through, an article. The authors have granted ASM a non-exclusive, world-wide license to publish the Supplemental Material files. Please contact the corresponding author directly for reuse.

## References

[1] Yin W, Xu SY, Wang YT, Zhang YL, Chou SH, Galperin MY, He J. 2021. Ways to control harmful biofilms: prevention, inhibition, and eradication. Crit Rev Microbiol 47:57–78. doi:10.1080/1040841X.2020.1842325 33356690 PMC7954276

[2] Yaron S, Römling U. 2014. Biofilm formation by enteric pathogens and its role in plant colonization and persistence. Microb Biotechnol 7:496–516. doi:10.1111/1751-7915.12186 25351039 PMC4265070

[3] Soutourina OA, Bertin PN. 2003. Regulation cascade of flagellar expression in Gram-negative bacteria. FEMS Microbiol Rev 27:505–523. doi:10.1016/S0168-6445(03)00064-0 14550943

[4] Kimkes TEP, Heinemann M. 2020. How bacteria recognise and respond to surface contact. FEMS Microbiol Rev 44:106–122. doi:10.1093/femsre/fuz029 31769807 PMC7053574

[5] Harshey RM. 2003. Bacterial motility on a surface: many ways to a common goal. Annu Rev Microbiol 57:249–273. doi:10.1146/annurev.micro.57.030502.091014 14527279

[6] Josenhans C, Suerbaum S. 2002. The role of motility as a virulence factor in bacteria. Int J Med Microbiol 291:605–614. doi:10.1078/1438-4221-00173 12008914

[7] Ottemann KM, Miller JF. 1997. Roles for motility in bacterial-host interactions. Mol Microbiol 24:1109–1117. doi:10.1046/j.1365-2958.1997.4281787.x 9218761

[8] Chen Y, Zhou J, Lv M, Liang Z, Parsek MR, Zhang L-H. 2020. Systematic analysis of c-di-GMP signaling mechanisms and biological functions in Dickeya zeae EC1. mBio 11:e02993-20. doi:10.1128/mBio.02993-20 PMC7733949 33262261

[9] Shi Z, Wang Q, Li Y, Liang Z, Xu L, Zhou J, Cui Z, Zhang LH. 2019. Putrescine is an intraspecies and interkingdom cell-cell communication signal modulating the virulence of Dickeya zeae. Front Microbiol 10:1950. doi:10.3389/fmicb.2019.01950 31497009 PMC6712546

[10] Gevrekci AÖ. 2017. The roles of polyamines in microorganisms. World J Microbiol Biotechnol 33:204. doi:10.1007/s11274-017-2370-y 29080149

[11] Boyle SM, Markham GD, Hafner EW, Wright JM, Tabor H, Tabor CW. 1984. Expression of the cloned genes encoding the putrescine biosynthetic enzymes and methionine adenosyltransferase of Escherichia coli (speA, speB, speC and metK). Gene 30:129–136. doi:10.1016/0378-1119(84)90113-6 6392022

[12] Tabor CW, Tabor H, Xie QW. 1986. Spermidine synthase of Escherichia coli: localization of the speE gene. Proc Natl Acad Sci USA 83:6040–6044. doi:10.1073/pnas.83.16.6040 3526348 PMC386433

[13] Tabor CW, Tabor H. 1987. The speEspeD operon of Escherichia coli. Formation and processing of a proenzyme form of S-adenosylmethionine decarboxylase. J Biol Chem 262:16037–16040. 3316212

[14] Chattopadhyay MK, Tabor CW, Tabor H. 2009. Polyamines are not required for aerobic growth of Escherichia coli: preparation of a strain with deletions in all of the genes for polyamine biosynthesis. J Bacteriol 191:5549–5552. doi:10.1128/JB.00381-09 19542271 PMC2725612

[15] Park YK, Bearson B, Bang SH, Bang IS, Foster JW. 1996. Internal pH crisis, lysine decarboxylase and the acid tolerance response of Salmonella typhimurium. Mol Microbiol 20:605–611. doi:10.1046/j.1365-2958.1996.5441070.x 8736539

[16] Kurihara S, Suzuki H, Tsuboi Y, Benno Y. 2009. Dependence of swarming in Escherichia coli K-12 on spermidine and the spermidine importer. FEMS Microbiol Lett 294:97–101. doi:10.1111/j.1574-6968.2009.01552.x 19493013

[17] Chattopadhyay MK, Tabor CW, Tabor H. 2003. Polyamines protect Escherichia coli cells from the toxic effect of oxygen. Proc Natl Acad Sci USA 100:2261–2265. doi:10.1073/pnas.2627990100 12591940 PMC151328

[18] Huang Y, Chen Y, Zhang L-H. 2020. The roles of microbial cell-cell chemical communication systems in the modulation of antimicrobial resistance. Antibiotics (Basel) 9:779. doi:10.3390/antibiotics9110779 33171916 PMC7694446

[19] Zhou L, Wang J, Zhang L-H. 2007. Modulation of bacterial type III secretion system by a spermidine transporter dependent signaling pathway. PLoS One 2:e1291. doi:10.1371/journal.pone.0001291 18074016 PMC2110884

[20] Lin Q, Wang H, Huang J, Liu Z, Chen Q, Yu G, Xu Z, Cheng P, Liang Z, Zhang L-H. 2022. Spermidine is an intercellular signal modulating T3SS expression in Pseudomonas aeruginosa. Microbiol Spectr 10:e00644-22. doi:10.1128/spectrum.00644-22 35435755 PMC9241758

[21] Hecht GB, Newton A. 1995. Identification of a novel response regulator required for the swarmer-to-stalked-cell transition in Caulobacter crescentus. J Bacteriol 177:6223–6229. doi:10.1128/jb.177.21.6223-6229.1995 7592388 PMC177463

[22] Merkel TJ, Barros C, Stibitz S. 1998. Characterization of the bvgR locus of Bordetella pertussis. J Bacteriol 180:1682–1690. doi:10.1128/JB.180.7.1682-1690.1998 9537363 PMC107078

[23] Galperin MY, Natale DA, Aravind L, Koonin EV. 1999. A specialized version of the HD hydrolase domain implicated in signal transduction. J Mol Microbiol Biotechnol 1:303–305. 10943560 PMC5330256

[24] Hengge R. 2021. High-specificity local and global c-di-GMP signaling. Trends Microbiol 29:993–1003. doi:10.1016/j.tim.2021.02.003 33640237

[25] Valentini M, Filloux A. 2019. Multiple roles of c-di-GMP signaling in bacterial pathogenesis. Annu Rev Microbiol 73:387–406. doi:10.1146/annurev-micro-020518-115555 31500536

[26] Cheang QW, Xin L, Chea RYF, Liang ZX. 2019. Emerging paradigms for PilZ domain-mediated C-di-GMP signaling. Biochem Soc Trans 47:381–388. doi:10.1042/BST20180543 30710060

[27] Paul K, Nieto V, Carlquist WC, Blair DF, Harshey RM. 2010. The c-di-GMP binding protein YcgR controls flagellar motor direction and speed to affect chemotaxis by a “backstop brake” mechanism. Mol Cell 38:128–139. doi:10.1016/j.molcel.2010.03.001 20346719 PMC2929022

[28] Johnson JG, Murphy CN, Sippy J, Johnson TJ, Clegg S. 2011. Type 3 fimbriae and biofilm formation are regulated by the transcriptional regulators MrkHI in Klebsiella pneumoniae. J Bacteriol 193:3453–3460. doi:10.1128/JB.00286-11 21571997 PMC3133326

[29] Sebghati TA, Korhonen TK, Hornick DB, Clegg S. 1998. Characterization of the type 3 fimbrial adhesins of Klebsiella strains. Infect Immun 66:2887–2894. doi:10.1128/IAI.66.6.2887-2894.1998 9596764 PMC108286

[30] Wang F, He Q, Su K, Gao F, Huang Y, Lin Z, Zhu D, Gu L. 2016. The PilZ domain of MrkH represents a novel DNA binding motif. Protein Cell 7:766–772. doi:10.1007/s13238-016-0317-y 27650952 PMC5055493

[31] Zhang JX, Lin BR, Shen HF, Pu XM. 2013. Genome sequence of the Banana pathogen Dickeya zeae strain MS1, which causes bacterial soft rot. Genome Announc 1:e00317-13. doi:10.1128/genomeA.00317-13 23766402 PMC3707573

[32] Hussain MBBM, Zhang H-B, Xu J-L, Liu Q, Jiang Z, Zhang L-H. 2008. The acyl-homoserine lactone-type quorum-sensing system modulates cell motility and virulence of Erwinia chrysanthemi pv. zeae. J Bacteriol 190:1045–1053. doi:10.1128/JB.01472-07 18083823 PMC2223575

[33] Samson R, Legendre JB, Christen R, Saux MF-L, Achouak W, Gardan L. 2005. Transfer of Pectobacterium chrysanthemi (Burkholder et al. 1953) Brenner et al. 1973 and Brenneria paradisiaca to the genus Dickeya gen. nov. as Dickeya chrysanthemi comb. nov. and Dickeya paradisiaca comb. nov. and delineation of four novel species, Dickeya dadantii sp. nov., Dickeya dianthicola sp. nov., Dickeya dieffenbachiae sp. nov. and Dickeya zeae sp. nov. Int J Syst Evol Microbiol 55:1415–1427. doi:10.1099/ijs.0.02791-0 16014461

[34] Wang X, He S-W, Guo H-B, Han J-G, Thin kyu kyu, Gao J, Wang Y, Zhang X-X. 2020. Dickeya oryzae sp. nov., isolated from the roots of rice. Int J Syst Evol Microbiol 70:4171–4178. doi:10.1099/ijsem.0.004265 32552985

[35] Zhou J, Zhang H, Wu J, Liu Q, Xi P, Lee J, Liao J, Jiang Z, Zhang L-H. 2011. A novel multidomain polyketide synthase is essential for zeamine production and the virulence of Dickeya zeae. Mol Plant Microbe Interact 24:1156–1164. doi:10.1094/MPMI-04-11-0087 21899437

[36] Hu M, Li J, Chen R, Li W, Feng L, Shi L, Xue Y, Feng X, Zhang L, Zhou J. 2018. Dickeya zeae strains isolated from rice, banana and clivia rot plants show great virulence differentials. BMC Microbiol 18:136. doi:10.1186/s12866-018-1300-y 30336787 PMC6194671

[37] Yang CH, Gavilanes-Ruiz M, Okinaka Y, Vedel R, Berthuy I, Boccara M, Chen JWT, Perna NT, Keen NT. 2002. hrp genes of Erwinia chrysanthemi 3937 are important virulence factors. Mol Plant Microbe Interact 15:472–480. doi:10.1094/MPMI.2002.15.5.472 12036278

[38] Yap MN, Yang CH, Barak JD, Jahn CE, Charkowski AO. 2005. The Erwinia chrysanthemi type III secretion system is required for multicellular behavior. J Bacteriol 187:639–648. doi:10.1128/JB.187.2.639-648.2005 15629935 PMC543537

[39] Chen Y, Lv M, Liao L, Gu Y, Liang Z, Shi Z, Liu S, Zhou J, Zhang L. 2016. Genetic modulation of c-di-GMP turnover affects multiple virulence traits and bacterial virulence in rice pathogen Dickeya zeae. PLoS One 11:e0165979. doi:10.1371/journal.pone.0165979 27855163 PMC5113947

[40] Liu F, Hu M, Zhang Z, Xue Y, Chen S, Hu A, Zhang L-H, Zhou J. 2022. Dickeya manipulates multiple quorum sensing systems to control virulence and collective behaviors. Front Plant Sci 13:838125. doi:10.3389/fpls.2022.838125 35211146 PMC8860905

[41] De N, Navarro MVAS, Raghavan RV, Sondermann H. 2009. Determinants for the activation and autoinhibition of the diguanylate cyclase response regulator WspR. J Mol Biol 393:619–633. doi:10.1016/j.jmb.2009.08.030 19695263 PMC2760619

[42] Rao F, Yang Y, Qi Y, Liang ZX. 2008. Catalytic mechanism of cyclic di-GMP-specific phosphodiesterase: a study of the EAL domain-containing RocR from Pseudomonas aeruginosa. J Bacteriol 190:3622–3631. doi:10.1128/JB.00165-08 18344366 PMC2394985

[43] Chen Y, Lv M, Liang Z, Liu Z, Zhou J, Zhang LH. 2022. Cyclic di-GMP modulates sessile-motile phenotypes and virulence in Dickeya oryzae via two PilZ domain receptors. Mol Plant Pathol 23:870–884. doi:10.1111/mpp.13200 35254732 PMC9104268

[44] Ogawara H, Kasama H, Nashimoto K, Ohtsubo M, Higashi K, Urabe H. 1993. Cloning, sequence and expression of the argG gene from Streptomyces lavendulae. Gene 125:91–96. doi:10.1016/0378-1119(93)90751-n 8449418

[45] Rosen BP. 1971. Basic amino acid transport in Escherichia coli. J Biol Chem 246:3653–3662. 4931309

[46] Wei YH, Newman EB. 2002. Studies on the role of the metK gene product of Escherichia coli K-12. Mol Microbiol 43:1651–1656. doi:10.1046/j.1365-2958.2002.02856.x 11952912

[47] Hickman JW, Harwood CS. 2008. Identification of FleQ from Pseudomonas aeruginosa as a c-di-GMP-responsive transcription factor. Mol Microbiol 69:376–389. doi:10.1111/j.1365-2958.2008.06281.x 18485075 PMC2612001

[48] Zorraquino V, García B, Latasa C, Echeverz M, Toledo-Arana A, Valle J, Lasa I, Solano C. 2013. Coordinated cyclic-di-GMP repression of Salmonella motility through YcgR and cellulose. J Bacteriol 195:417–428. doi:10.1128/JB.01789-12 23161026 PMC3554008

[49] Roth M. 1971. Fluorescence reaction for amino acids. Anal Chem 43:880–882. doi:10.1021/ac60302a020 5576608

[50] Ogden G, Foldi P. 1987. Amino acid analysis: an overview of current methods. LC GC 5:28–40.

[51] Shah P, Swiatlo E. 2008. A multifaceted role for polyamines in bacterial pathogens. Mol Microbiol 68:4–16. doi:10.1111/j.1365-2958.2008.06126.x 18405343

[52] Kashiwagi K, Igarashi K. 1988. Adjustment of polyamine contents in Escherichia coli. J Bacteriol 170:3131–3135. doi:10.1128/jb.170.7.3131-3135.1988 3290196 PMC211259

[53] Kurihara S, Oda S, Tsuboi Y, Kim HG, Oshida M, Kumagai H, Suzuki H. 2008. Gamma-glutamylputrescine synthetase in the putrescine utilization pathway of Escherichia coli K-12. J Biol Chem 283:19981–19990. doi:10.1074/jbc.M800133200 18495664

[54] Lee J, Sperandio V, Frantz DE, Longgood J, Camilli A, Phillips MA, Michael AJ. 2009. An alternative polyamine biosynthetic pathway is widespread in bacteria and essential for biofilm formation in Vibrio cholerae. J Biol Chem 284:9899–9907. doi:10.1074/jbc.M900110200 19196710 PMC2665113

[55] Sethi R, Chava SR, Bashir S, Castro ME. 2011. An improved high performance liquid chromatographic method for identification and quantization of polyamines as benzoylated derivatives. AJAC 02:456–469. doi:10.4236/ajac.2011.24055

[56] Miller WG, Leveau JH, Lindow SE. 2000. Improved gfp and inaZ broad-host-range promoter-probe vectors. Mol Plant Microbe Interact 13:1243–1250. doi:10.1094/MPMI.2000.13.11.1243 11059491

[57] Song JP, Zhou CW, Liu R, Wu XD, Wu D, Hu XJ, Ding Y. 2010. Expression and purification of recombinant arginine decarboxylase (speA) from Escherichia coli. Mol Biol Rep 37:1823–1829. doi:10.1007/s11033-009-9617-0 19603287

[58] Chen Y, Li Y, Zhu M, Lv M, Liu Z, Chen Z, Huang Y, Gu W, Liang Z, Chang C, Zhou J, Zhang LH, Liu Q. 2022. The GacA-GacS Type two-component system modulates the pathogenicity of Dickeya oryzae EC1 mainly by regulating the production of zeamines. Mol Plant Microbe Interact 35:369–379. doi:10.1094/MPMI-11-21-0292-R 35100009

