# Supplementary material for: The cyclic di-GMP receptor YcgR links the second messenger with the putrescine quorum sensing system in modulation of Dickeya oryzae motility
Source: mBio. 2025 May 30;16(7):e01016-25. doi: 10.1128/mbio.01016-25 (PMC12239582; doi:10.1128/mbio.01016-25)
Supplement: Supplemental material — Supplemental tables and figures. [file mbio.01016-25-s0001.docx]

**SUPPLEMENTAL MATERIAL**

**The cyclic di-GMP receptor YcgR links the second messenger with putrescine quorum sensing system in modulation of *Dickeya oryzae* motility**

Weihan Gu^a#^, Yufan Chen^b#^, Congcong Xie^c^, Zhongqiao Chen^a^, Huagui Gao^a^, Yaping Zhu^a^, Lian-hui Zhang^a*^, Lisheng Liao^a*^

^a^Guangdong Province Key Laboratory of Microbial Signals and Disease Control, Integrative Microbiology Research Center, South China Agricultural University, Guangzhou 510642, China

^b^Research Center of Chinese Herbal Resource Science and Engineering, Key Laboratory of Chinese Medicinal Resource From Lingnan, Ministry of Education, Joint Laboratory of National Engineering Research Center for the Pharmaceutics of Traditional Chinese Medicines, Guangzhou University of Chinese Medicine, Guangzhou, 510006, People's Republic of China.

^c^School of Biological and Environmental Engineering, Jingdezhen University, Jingdezhen, 333000, China

^*^Correspondence: Lian-Hui Zhang, [lhzhang01@scau.edu.cn](mailto:lhzhang01@scau.edu.cn) or

Lisheng Liao, [lishengliao@scau.edu.cn](mailto:lishengliao@scau.edu.cn)

^#^Contributed equally to this work.

**Contents**

TABLE S1 Strains and plasmids used in this study.

TABLE S2 Primers used in this study.

FIG S1-S7

**Table S1. Strains and plasmids used in this study.**

| **Strain or plasmid** | **Relevant characteristics^a^** | **References of source** |
| --- | --- | --- |
| **Strains** |  |  |
| *Dickeya oryzae* |  |  |
| EC1 | Wild-type strain, Pm^r^ | Lab collection |
| 7ΔPDE | Deletion of all c-di-GMP degradation genes of EC1, Pm^r^ | Lab collection |
| 7ΔPDEΔ*ycgR* | *W909_08750* gene deletion of 7ΔPDE, Pm^r^ | Lab collection |
| Δ*ycgR* | *W909_08750* gene deletion mutant, Pm^r^ | Lab collection |
| 15ΔDGC | Deletion of all c-di-GMP synthase genes of EC1, Pm^r^ | Lab collection |
| Δ*speA* | *W909_17465* gene deletion mutant, Pm^r^ | This study |
| Δ*argG* | *W909_00315* gene deletion mutant, Pm^r^ | This study |
| Δ*artP* | *W909_08445* gene deletion mutant, Pm^r^ | This study |
| Δ*metK* | *W909_17475* gene deletion mutant, Pm^r^ | This study |
| Δ*speA*Δ*ycgR* | *W909_08750* gene deletion of Δ*speA*, Pm^r^ | This study |
| Δ*speA*Δ*potF*Δ*plaP* | *W909_08395* and *W909_08760* gene deletion of Δ*speA*, Pm^r^ | This study |
| 15ΔDGCΔ*speA* | *W909_17465* gene deletion of 15ΔDGC,Pm^r^ | This study |
| 7ΔPDEΔ*speA* | *W909_17465* gene deletion of 7ΔPDE,Pm^r^ | This study |
| Δ*ycgR*(*ycgR*) | Δ*ycgR* carrying pBBR1-ycgR vector, Ap^r^ | This study |
| 7ΔPDE(*rocR*) | 7ΔPDE carrying pBBR1-rocR vector, Ap^r^ | This study |
| 15ΔDGC(*wspR*) | 15ΔDGC carrying pBBR1-wspR vector, Ap | Lab collection |
| Δ*speA*Δ*potF*Δ*plaP*(*rocR*) | Δ*speA*Δ*potF*Δ*plaP* carrying pBBR1-rocR vector, Ap^r^ | This study |
| EC1(*ycgR*) | Wild-type strain carrying pBBR1-ycgR vector, Pm^r^ | This study |
| *Escherichia coli* |  |  |
| K12 CC118 | *gyrA*，*recA*，*λ pir* | Lab collection |
| DH5α | *deoR*, *recA*, *endA*, *hsdR*, *supE*, *thi*, *gyrA*, *relA* | Lab collection |
| BL21 | *B F^–^ ompT gal dcm lon hsdS_B_(r_B_^–^m_B_^–^) [malB^+^]_K-12_(λ^S^)* | Lab collection |
| **Plasmids** |  |  |
| pKNG101 | Suicide vector; Str^r^, SacB, mobRK2, oriR6K (pir-minus) | Lab collection |
| pKNG-*ycgR* | *W909_08750* knock-out fragment ligated on pKNG101 | This study |
| pKNG-*speA* | *W909_17465* knock-out fragment ligated on pKNG101 | This study |
| pKNG-*potF* | *W909_08395* knock-out fragment ligated on pKNG101 | This study |
| PKNG-*plaP* | *W909_08760* knock-out fragment ligated on pKNG101 | This study |
| pKNG-*argG* | *W909_00315* knock-out fragment ligated on pKNG101 | This study |
| pKNG-*artP* | *W909_08445* knock-out fragment ligated on pKNG101 | This study |
| pKNG-*metK* | *W909_17475* knock-out fragment ligated on pKNG101 | This study |
| pBBR1MCS-4 | Expression vector contains a *lacZ* promoter, Ap^r^ | Lab collection |
| pBBR1-*ycgR* | pBBR1-MCS4 carries the coding region of *W909_08750* at down-stream of lac promoter, Ap^r^ | This study |
| pBBR1-*rocR* | pBBR1-MCS4 carries the coding region of PA3947 at down-stream of lac promoter, Ap^r^ | Lab collection |
| pPROBE-NT | Promoterless *gfp* transcriptional reporter plasmid, Kan^r^ | Lab collection |
| pDdgcA_gfp_ | *gfp* transcriptional fusion with upstream region of *ddcgA* in EC1 | This study |
| pDpdeA_gfp_ | *gfp* transcriptional fusion with upstream region of *dpdeA* in EC1 | This study |
| pDpdeB_gfp_ | *gfp* transcriptional fusion with upstream region of *dpdeB* in EC1 | This study |
| pSpeA_gfp_ | *gfp* transcriptional fusion with upstream region of *speA* in EC1 | This study |
| pSpeC_gfp_ | *gfp* transcriptional fusion with upstream region of *speC* in EC1 | This study |
| pGEX-6P-1 | Protein expression vector containing GST tag, Ap^r^ | Lab collection |
| pGEX-YcgR | pGEX-6P-1 carries the coding region of *W909_08750* at down-stream of GST tag, Ap^r^ | This study |
| pET-32a | Protein expression vector containing HIS tag, Ap^r^ | Lab collection |
| pET-SpeA | pET-32a carries the coding region of *W909_16940* at down-stream of HIS tag, Ap^r^ | This study |
| pBT | Two-hybrid system bait plasmid containing the *cat* gene, p15A origin of replication and λ cI ORF | Lab collection |
| pBT-YcgR1 | pBT containing *ycgR* of EC1 | This study |
| pBT-YcgR2 | pBT containing *ycgR* of *e.coli* | This study |
| pTRG | Two-hybrid system target plasmid containing the *tet* gene, ColE1 origin of replication, and RNA polymerase α subunit ORF | Lab collection |
| pTRG-FliG1 | pTRG containing *fliG* of EC1 | This study |
| pTRG-FliG2 | pBT containing *fliG* of *e.coli* | This study |
| pTRG-SpeA | pTRG containing *speA* of EC1 | This study |
| pTRG-ArgG | pTRG containing *argG* of EC1 | This study |
| pTRG-ArtP | pTRG containing *artP* of EC1 | This study |
| pTRG-MetK | pTRG containing *metK* of EC1 | This study |

**Abbreviations:** Ap^r^, ampicillin resistance; Pm^r^, polymyxinB resistance; Kan^r^, kanamycin resistance; Str^r^, streptomycin resistance;Cm^r^,chloramphenicol resistance.

**Table S2. Primers used in this study.**

| **Primer** | **Primer sequence (5’-3’)** |
| --- | --- |
| **For deletion** | |
| SpeA-1 | cccctgcaggtcgacggatccCTTTTCTTCTGACGCCACTGC |
| SpeA-2 | ttctaacgagagaGGCGATCCCCTTCTTCCA |
| SpeA-3 | gatcgccTCTCTCGTTAGAAAAGGTTGAGTGC |
| SpeA-4 | cggactatagactatactagtCGGGACAACAACGCCGCC |
| PotF-1 | cccctgcaggtcgacggatccTACGTTATGGATCAATAGCTGGTCA |
| PotF-2 | CCGTTCCTTCCTCCATACCAG |
| PotF-3 | tggtatggaggaaggaacggCCGCTGTCCTTGACTCCACTT |
| PotF-4 | cggactatagactatactagtCTGCACAAATTTACCGCGATT |
| PlaP-1 | cccctgcaggtcgacggatccTGGCTAGCTGGGGCAAAAT |
| PlaP-2 | cctgataTACGCAAACCTCCTTTACCGA |
| PlaP-3 | ggaggtttgcgtaTATCAGGTGATGAGAACGGGCT |
| PlaP-4 | cggactatagactatactagtAGATATTCAGCCACTACTGGCGC |
| ArgG-1 | cccctgcaggtcgacggatccGCGGTGGTGGGGGGATAT |
| ArgG-2 | caggacgcgcCATAACTATTAGTCCCTGCTTGATTTC |
| ArgG-3 | aatagttatgGCGCGTCCTGAAGGACATG |
| ArgG-4 | cggactatagactatactagtCGTGATATTCATAAGTAACACTATATATTACGA |
| ArtP-1 | cccctgcaggtcgacggatccGTTGTTCGAGGCAGCCTATTATT |
| ArtP-2 | gcatgcgtaCATCAAACCGTCCTTCTCTTCAA |
| ArtP-3 | acggtttgatgTACGCATGCCTCACCGTCC |
| ArtP-4 | cggactatagactatactagtCGGTGTTAGTGGCGAAGAACG |
| MetK-1 | cccctgcaggtcgacggatccAATCCTATCACCTCCACGCTCG |
| MetK-2 | catgtcagaaaagcggaGAGTTTCTTTACCTTATAAGCTAAAGCC |
| MetK-3 | ctcTCCGCTTTTCTGACATGCCA |
| MetK-4 | cggactatagactatactagtATTCCAACGTCACCTGCAGG |
| YcgR-1 | cccctgcaggtcgacggatccTTTCACACTATCCTGCCGCTT |
| YcgR-2 | tatctgtttgacttcgcCCCCACCGTCGATTGAATG |
| YcgR-3 | gggGCGAAGTCAAACAGATAGAGCGC |
| YcgR-4 | cggactatagactatactagtAGTCCCATCATTACCACTGGCA |
| PKNG-F | GCCATCAAACCACGTCAAAT |
| PKNG-R | AACCAAGCCTATGCCTACAG |
| **For *in trans* expression** | |
| CYcgR-1 | gataagcttgatatcgaattcATGGATGTAGTGGATGACAATATGAAA |
| CYcgR-2 | cgctctagaactagtggatccATAGTACAGCACGTCAGGTATCGC |
| MCS-F | TCTTCGCTATTACGCCAGCT |
| MCS-R | GGCTCGTATGTTGTGTGGAA |
| **For protein expression** | |
| pGEX-(ycgR)-1 | ttccaggggcccctgggatccATGGATGTAGTGGATGACAATATGAAA |
| pGEX-(ycgR)-2 | ctcgagtcgacccgggaattcAACCGACGCGATAACGGTG |
| pGEX-F | GGGCTGGCAAGCCACGTTTGGTG |
| pGEX-R | CCGGGAGCTGCATGTGTCAGAGG |
| pET-(speA)-1 | gccatggctgatatcggatccATGTCTGACGATATGATTCAACCG |
| pET-(speA)-2 | ctcgagtgcggccgcaagcttCTCGTCTTCCAGATAAGTGTAACCG |
| pET-F | CATTCTTCTGGTCTGGTG |
| pET-R | CTCAAGACCCGTTTAGAG |
| **For bacterial two-hybrid assay** | |
| pB-ycgR-1(e.coli) | tggcgcggccgcatcgaattcCGTGAGTCATTACCATGAGCAGTT |
| pB-ycgR-2(e.coli) | aattaattaactcgaggatccTCAGTCGCGCACTTTGTCC |
| pB-ycgR-1(EC1) | tggcgcggccgcatcgaattcGATGGATGTAGTGGATGACAATATGA |
| pB-ycgR-2(EC1) | aattaattaactcgaggatccTCAACGCAGGCGTTTGCG |
| pT-fliG-1(e.coli) | gatccgcggccgcaagaattcTCATGAGTAACCTGACAGGCACC |
| pT-fliG-2(e.coli) | ttaattaattaattactcgagTCAGACATAGGTATCCTCGCCG |
| pT-speA-1(EC1) | gatccgcggccgcaagaattcCCATGTCTGACGATATGATTCAACC |
| pT-speA-2(EC1) | ttaattaattaattactcgagCTCGTCTTCCAGATAAGTGTAACCG |
| pT-argG-1(EC1) | gatccgcggccgcaagaattcTTATGACGACGATTTTGAAACATCT |
| pT-argG-2(EC1) | ttaattaattaattactcgagTTTCGCGGACTTATCCGGC |
| pT-artP-1(EC1) | gatccgcggccgcaagaattcTGATGATTTTCCTGAAAAATGTTTCT |
| pT-artP-2(EC1) | ttaattaattaattactcgagATGCAGGATTTTAGCCAAAAAGTC |
| pT-metK-1(EC1) | gatccgcggccgcaagaattcTCATGGCTAAACACCTTTTTACATC |
| pT-metK-2(EC1) | ttaattaattaattactcgagTTTCAGGCCTGCGGCATC |
| pBT-F | TCCGTTGTGGGGAAAGTTATC |
| pBT-R | GGGTAGCCAGCAGCATCC |
| pTRG-F | TGGCTGAACAACTGGAAGCT |
| pTRG-R | ATTCGTCGCCCGCCATAA |
| **For qPCR** | |
| DdgcA-Q1 | TATGTCGCTGATAAGATTC |
| DdgcA-Q2 | CGTTAATGGCTAATGTCA |
| DpdeA-Q1 | TTCATTATGCTGCTACAA |
| DpdeA-Q2 | TACAGTAAGTGGCTATCA |
| DpdeB-Q1 | ACTGCTATCGTTTCTTTA |
| DpdeB-Q2 | TTGTTATTGACCATATTGAC |
| speA-Q1 | CGATATGCTGGAATATGT |
| speA-Q2 | TAAGTGTAACCGTATAACC |
| speC-Q1 | GACGGCACTATCTATAAC |
| speC-Q2 | GATGAACTGCTCATATCC |
| metK-Q1 | CGAAGGACATCCTGATAA |
| metK-Q2 | CACCAACTAACACCATAC |
| artP-Q1 | AATGTGAACAAGTGGTAT |
| artP-Q2 | GGATTGTGGTTGATTTAC |
| argG-Q1 | GACAACCTGACATACAAA |
| argG-Q2 | ATATCCAGATTACGCATC |
| YcgR(qPCR)-1 | TAGTGGATGACAATATGA |
| YcgR(qPCR)-2 | AGGATCTTACTGATGAAT |
| **For construction of fluorescence reporter genes** | |
| NT-ddgcA-1 | ggaattggggatcggaagcttGAGTAAGTCCTCTCGACTCCCGC |
| NT-ddgcA-1 | gagctcggtacccggggatccTATTATAATTTCATACACAATAACACTCTCCTC |
| NT-dpdeA-1 | ggaattggggatcggaagcttGTTTTCTTTCCTTTCATCAATCAGC |
| NT-dpdeA-2 | gagctcggtacccggggatccCACTATGTATTCCGCCATTGAAGG |
| NT-dpdeB-1 | ggaattggggatcggaagcttGCAGTCTCTCCGATGTATTCCC |
| NT-dpdeB-2 | gagctcggtacccggggatccTGCTGGGGCTCGACTCATG |
| NT-YcgR-1 | ggaattggggatcggaagcttACCTCGTCTATTATTGGCTAACAGC |
| NT-YcgR-2 | gagctcggtacccggggatccCTTGCCACGCCTGGCGTA |
| NT-speA-1 | ggaattggggatcggaagcttTGACTCAATCCTTTCCGCCA |
| NT-speA-2 | gagctcggtacccggggatccAATCATATCGTCAGACATGGCG |
| NT-speC-1 | ggaattggggatcggaagcttGAGTTTCTGTCACAATCGCTGCG |
| NT-speC-2 | gagctcggtacccggggatccAATTTTTAACTGTTTCATGTACCCAAA |
| NT-potF-1 | ggaattggggatcggaagcttTTACGTTATGGATCAATAGCTGGTC |
| NT-potF-2 | gagctcggtacccggggatccCCGTTCCTTCCTCCATACCAG |
| NT-plaP-1 | ggaattggggatcggaagcttTTAATTAACATCAAGCCTGATAAGTGTG |
| NT-plaP-2 | gagctcggtacccggggatccTGTCTCTCTCTATTCACTCTGCGC |
| NT-F | TTGGGGATCGGAAGCTT |
| NT-R | CACCTTCACCCTCTCCACTG |


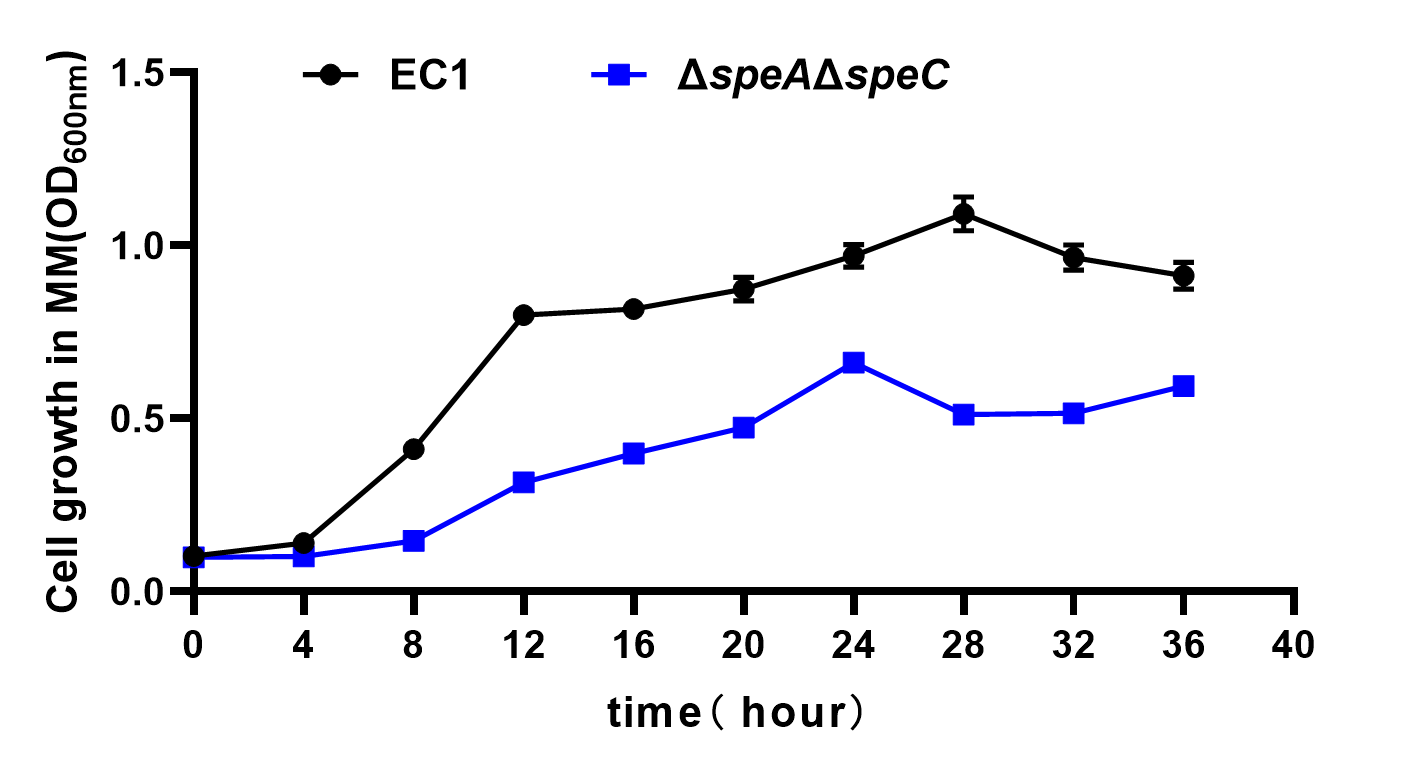


**FIG S1** **Growth curves of *Dickeya oryzae* EC1 and its derivatives in the MM medium.** Mutant ∆*speA*∆*speC* was generated by knocking out *speC* in the *speA*-deletion genetic background.


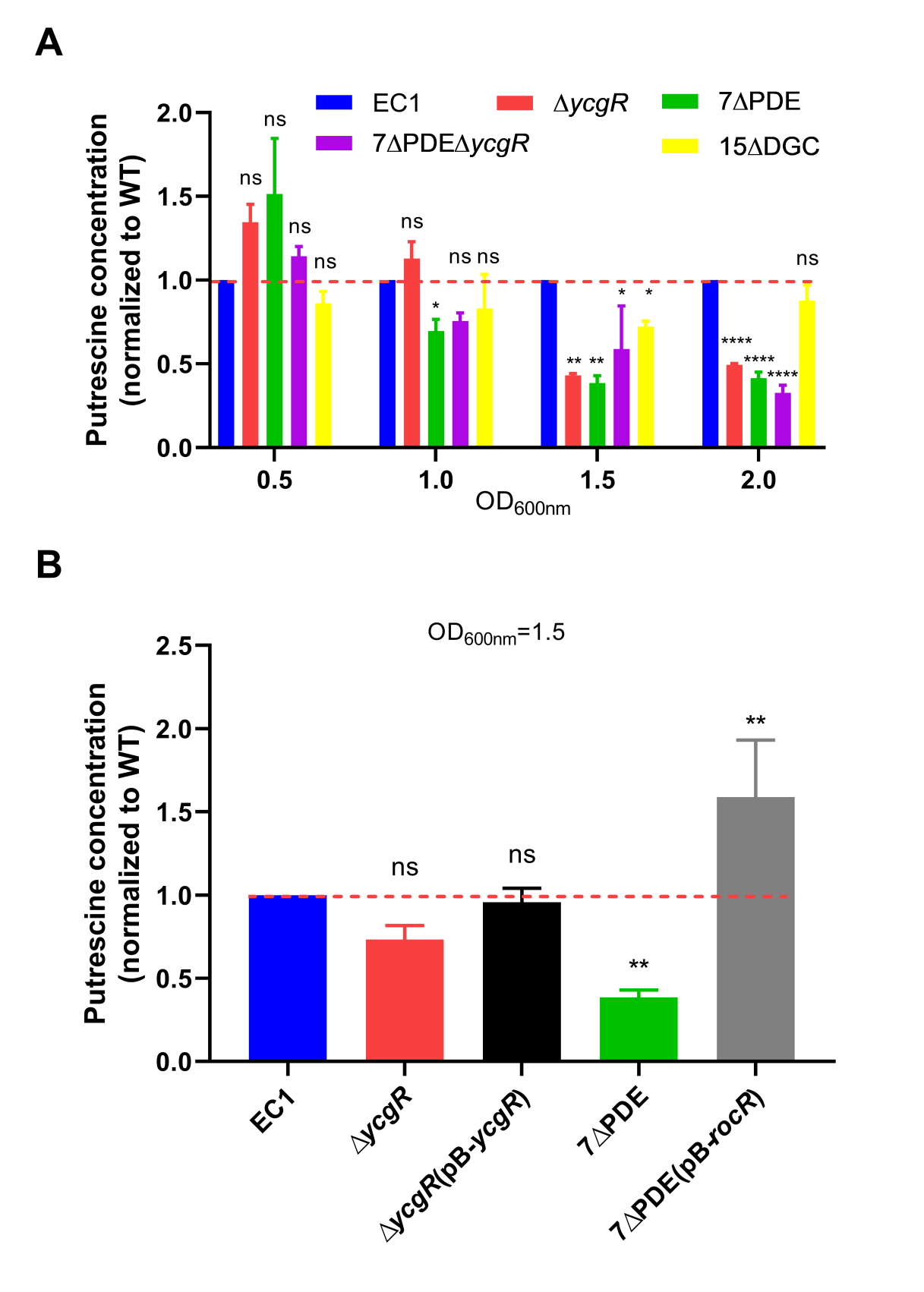


**FIG. S2 Cellular PUT levels in *D. oryzae* EC1 and its derivatives.** (A) Quantitative measurement of cellular PUT concentration of strain EC1 and the c-di-GMP signaling system-related mutants in LB medium. (B) Quantitative measurement of cellular PUT concentration of strain EC1, **∆***ycgR*, **∆***ycgR*(pB*-ycgR*)*,* 7**∆**PDE, 7**∆**PDE(pB-*rocR*) in LB medium. The data shown are the mean ± standard deviations (n = 3). Statistics significance: ****, *P* < 0.0001 **, *P* < 0.01; *, *P* < 0.05; ns, *P* > 0.05 (by one-way ANOVA with multiple comparisons). Dotted line indicates the PUT level of wild-type EC1.


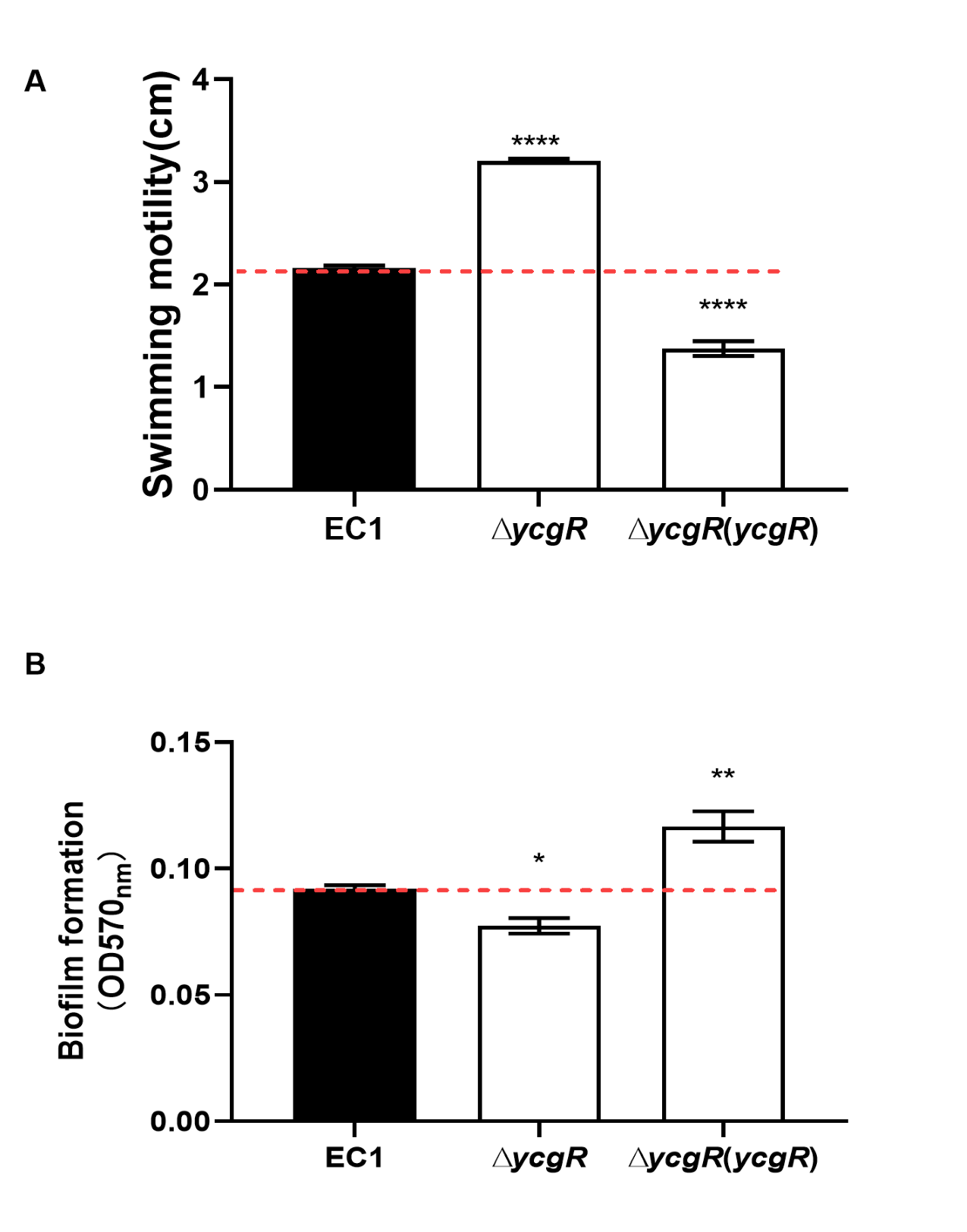


**FIG. S3 Deletion of the c-di-GMP receptor gene *ycgR* altered the swimming motility and biofilm formation of *D. oryzae* EC1.** Deletion of the c-di-GMP receptor resulted in increased swimming motility (A) and decreased biofilm formation (B). The data shown are the mean ± standard deviations (n = 3). Statistics significance: ****, *P* < 0.0001; **, *P* < 0.01, *, *P* < 0.05 (by one-way ANOVA with multiple comparisons).


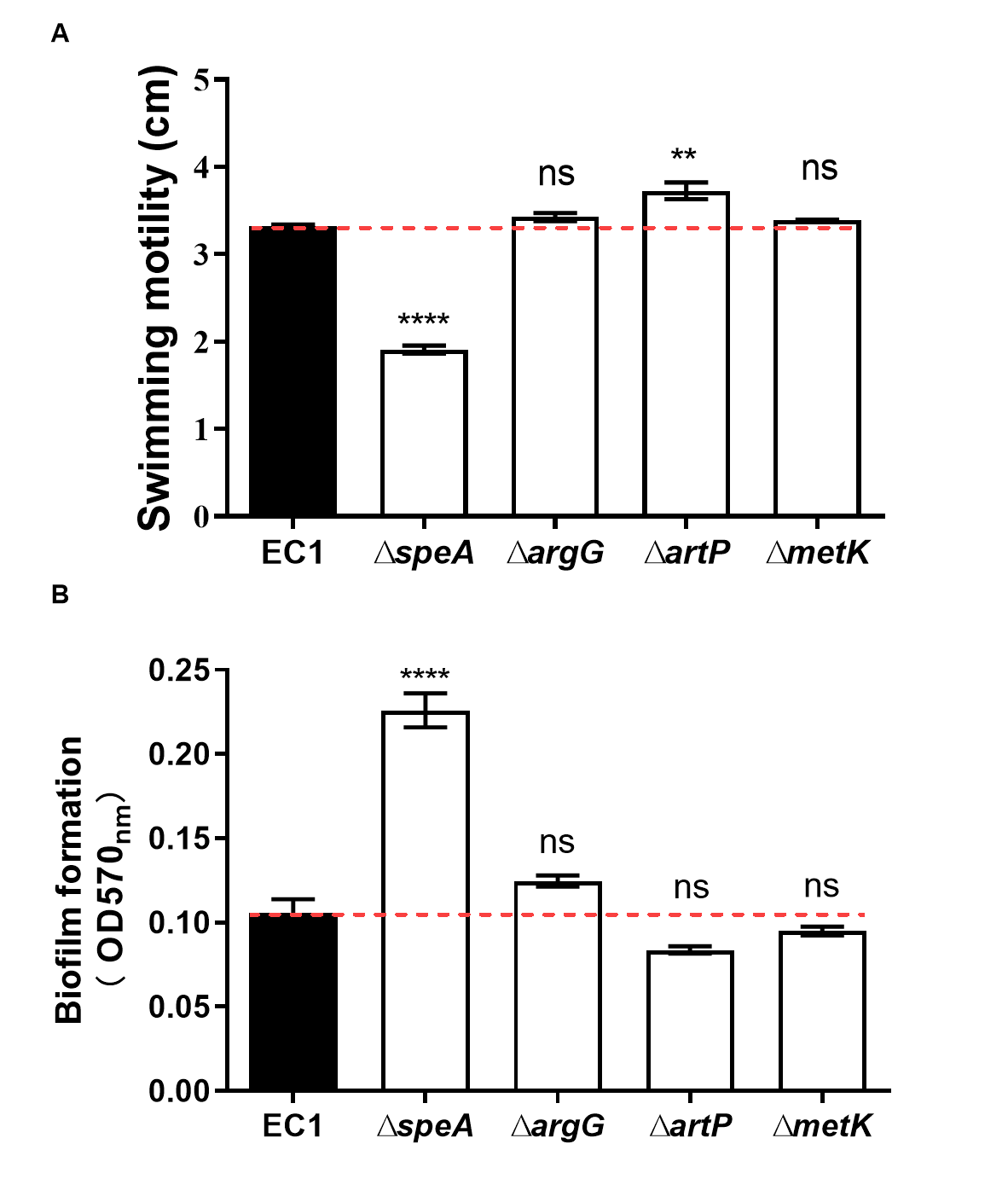


**FIG. S4 Validation of the roles of four YcgR-interacting proteins in regulation of swimming motility (A) and biofilm formation (B).** SpeA, biosynthetic arginine decarboxylase; ArtP, arginine ABC transporter ATP-binding protein; ArgG, argininosuccinate synthase; MetK, S-adenosylmethionine synthase. The data shown are the mean ± standard deviations (n = 3). Statistics significance: ****, *P* < 0.0001; **, *P* < 0.01, ns, *P* > 0.05 (by one-way ANOVA with multiple comparisons).


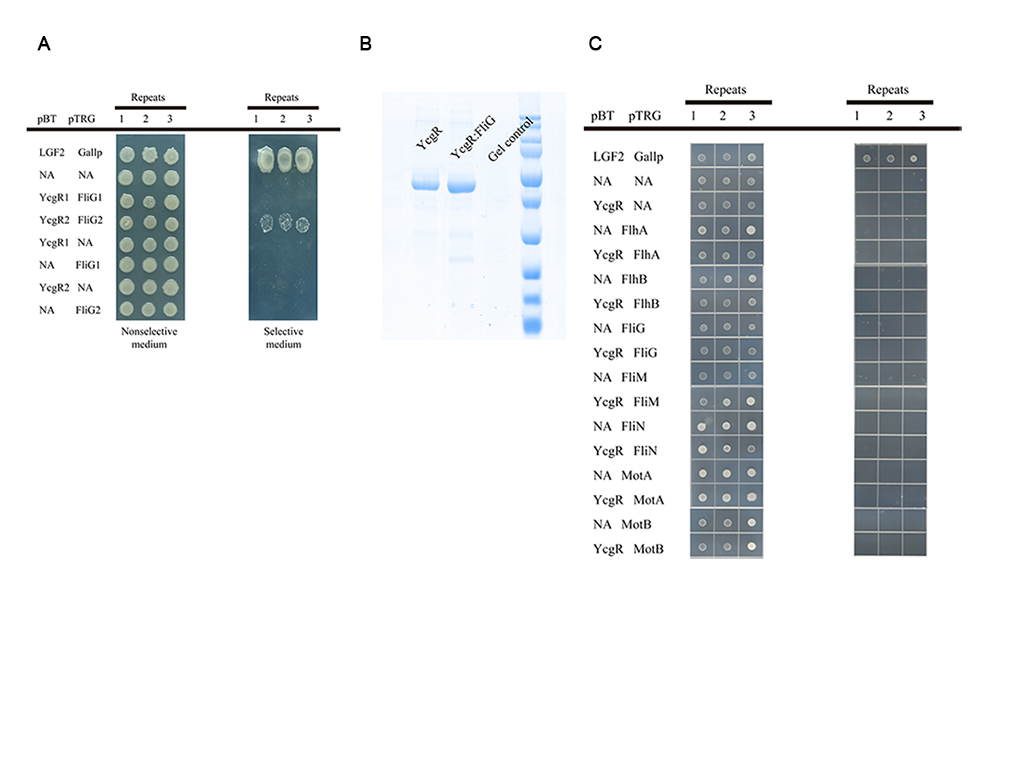


**FIG. S5 YcgR cannot interact with bacterial flagellar-associated proteins.** (A) Bacterial two-hybrid analysis. (B) Protein pull-down analysis. (C) Bacterial two-hybrid analysis of YcgR and other flagellar proteins from *D. oryzae* EC1. All the proteins are from *D. oryzae* EC1 except that YcgR2 and FilG2 from *E. coli* were used as controls in this experiment.

**
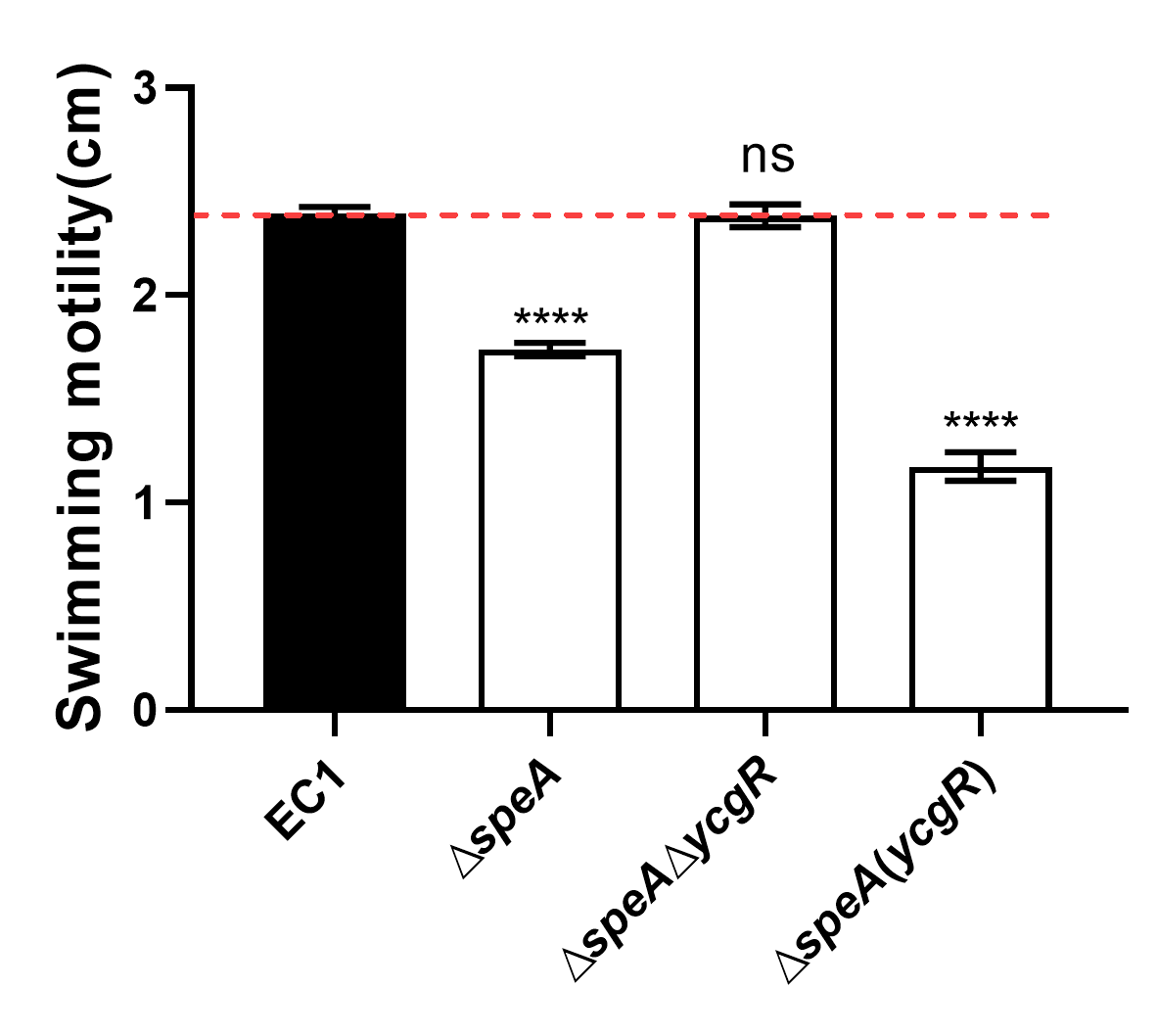
**

**FIG S6 Swimming motility assays after deletion and overexpression of *ycgR* gene, respectively, in the background of ∆*speA.*** The data shown are the mean ± standard deviations (n = 3). Statistics significance: ****, *P* < 0.0001; ns, *P* > 0.05 (by one-way ANOVA with multiple comparisons).

**
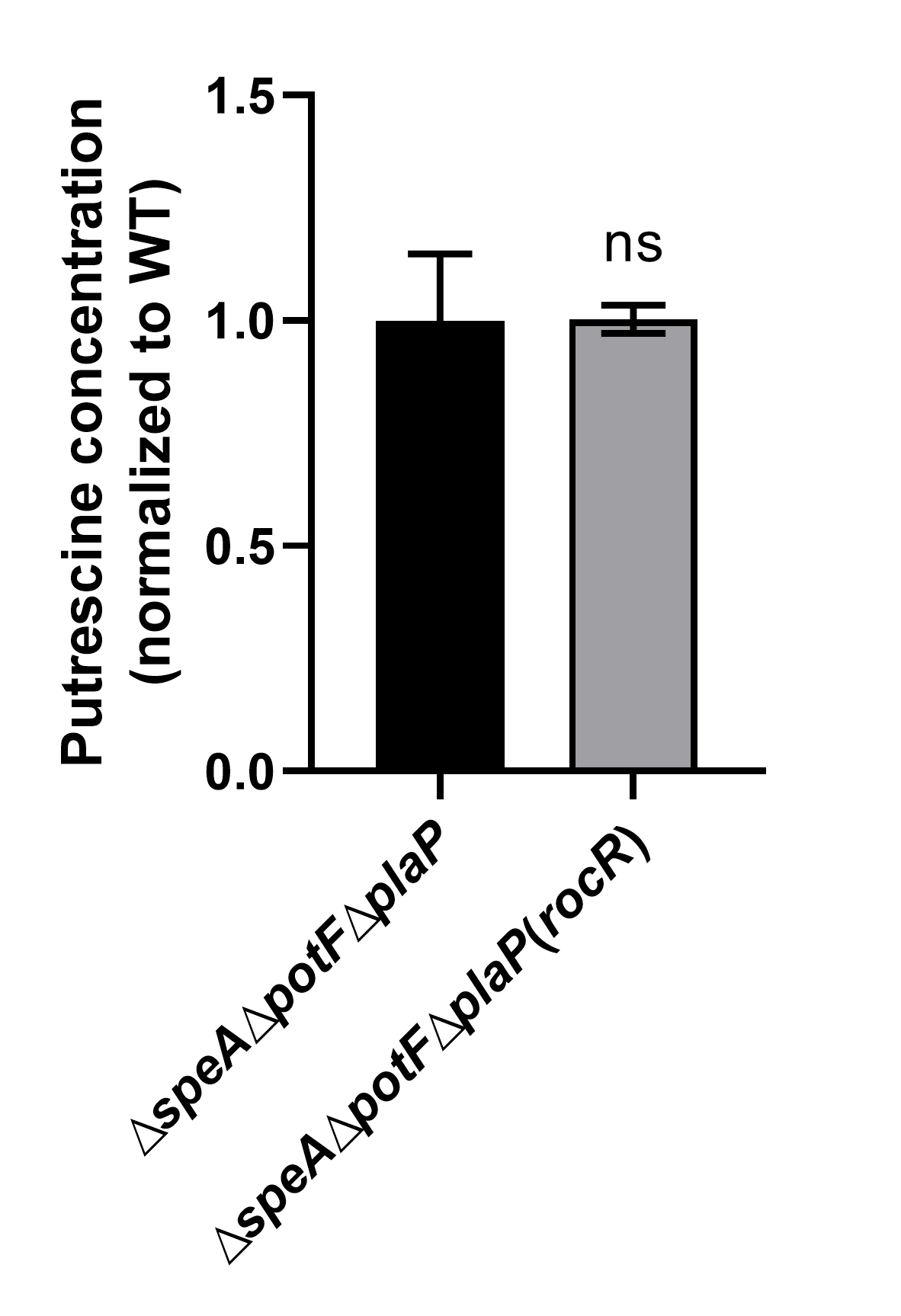
**

**FIG. S7 Cellular PUT levels in strains ∆*speA*∆*potF*∆*plaP* and ∆*speA*∆*potF*∆*plaP(rocR*).** The data shown are the mean ± standard deviations (n = 3). Statistics significance: ns, *P* > 0.05 (by Student’s unpaired *t* test).
